# *Parafenestella varangrensis* sp. nov., a phomenin producing fungus from the Arctic

**DOI:** 10.1038/s41598-025-33070-y

**Published:** 2025-12-23

**Authors:** Sailesh Maharjan, Julie Marie Lesjø, Johan Isaksson, Kine Østnes Hansen, Jeanette Hammer Andersen, Espen Holst Hansen, Teppo Rämä

**Affiliations:** 1 Marbio, Norwegian College of Fishery Science (NFH), Faculty of Biosciences, Fisheries, and Economics , UiT-The Arctic University of Norway, 9037 Tromsø, Norway; 2 Department of Pharmacy (IFA), Faculty of Health Sciences, UiT-The Arctic University of Norway, 9037 Tromsø, Norway; 3 Department of Chemistry (IK), Faculty of Science and Technology, UiT-The Arctic University of Norway, 9037 Tromsø, Norway

**Keywords:** Antibacterial *α*-pyrone, Polyketide, Phomenin, *Parafenestella*, varangrensis, Marine-derived fungus, Biochemistry, Biotechnology, Microbiology

## Abstract

Driftwood-associated fungi are a less recognized source of secondary metabolites with diverse biological activities. The main aim of this study was to characterize a driftwood-associated *Parafenestella* (*Pleosporales*, *Ascomycota*) species and evaluate the antibacterial and antibiofilm activities of its secondary metabolites. The fungus, isolated from driftwood on Varanger peninsula in the Northeast of Norway, was identified in the genus *Parafenestella* and is described here as *P. varangrensis* sp. nov. using a polyphasic taxonomic approach, including a multilocus phylogenetic analysis. Secondary metabolites were isolated from the *P. varangrensis* extract using flash chromatography followed by preparative high-performance liquid chromatography-mass spectrometry (prep HPLC-MS). The structures of the isolated compounds were elucidated through spectroscopic analysis, including 1D- and 2D-NMR, as well as HR-MS. They were identified as the known α-pyrone-derived polyketides, phomenins A and B, and their antibacterial activity was evaluated. Phomenin A exhibited weak antibacterial activity against *Streptococcus agalactiae*. Our findings highlight the importance of exploring cryptic biodiversity in less-studied environments, such as driftwood-associated marine-derived fungi in the Arctic, to expand our knowledge of secondary metabolites and their bioactivities.

**Supplementary Information:**

The online version contains supplementary material available at 10.1038/s41598-025-33070-y.

## Introduction

The genus *Parafenestella* (*Dothideomycetes*,* Ascomycota*) was first described and included in the family of *Cucurbitariaceae* within the order *Pleosporales* with *P. pseudoplatani* as a type-species along with other species such as *P. mackenziei*, and *P. ostryae*^1^. Within a few years, eighteen species of *Parafenestella* have been discovered and described based on morphological and molecular evidence^2,3^. Most species are saprobic and/or necrotrophic, but some are fungicolous^1,4^. The genus *Parafenestella* is characterized by its sexual stages producing ostiolate ascomata that form on a basal stromatic structure, cylindrical asci, and pigmented muriform ascospores. The asexual stages develop pycnidia that contain one-celled conidia produced in phialides^1^. Like many other families in the phylum *Ascomycota*, the systematics within the family has recently been established, yet changes are due through new combinations and introduction of new species.

The major knowledge gap in the study of the Arctic marine fungal diversity is that many environments and microhabitats remain largely understudied. In our continuous search for bioactive metabolites from fungi inhabiting underexplored ecological niches in the cold marine waters of the North, untargeted mass spectrometry-based metabolite profiling of a cultured *Parafenestella* fungus led to the identification of phomenins. Phomenins are polypropionate pyrone polyketides that have been isolated from fungi such as *Phoma tracheiphila*^5^, *Leptosphaeria maculans*^6,7^, and *Alternaria infectoria*^8^ showing phytotoxic, cytotoxic, and insecticidal effects. In nature, pyrones occur in two isomeric forms, *α*-pyrone (2-pyrone) or *γ*-pyrone (4-pyrone), which differ in the position of the carbonyl group relative to the ring oxygen^9,10^. Among the two isomeric forms, *α*-pyrones are prevalent in diverse natural products isolated from plants^11^, actinobacteria^12^ and fungi^13^. Examples of *α*-pyrone-containing natural products include isocoumarins^14^, meroterpenoids^15^, polyketides^16^, macrocyclics^17^, and heterocycles^18^.

Moreover, monocyclic *α*-pyrones with side chains of varying lengths represent an important subclass of natural products that are known for their wide range of biological activities. Several alkenylated or alkylated *α*-pyrones that are structurally similar to the phomenins have shown strong antibacterial effects. Examples of antibacterial pyrone derivatives with high structural similarity to the phomenins include nipyrone A-D^13^, and pseudopyronine A-C^19^. None of these compounds have been isolated from *Parafenestella* species. Therefore, this paper describes a previously undescribed species of *Parafenestella* isolated from driftwood sampled in northeastern Norway, together with isolation and investigation of the antibacterial activity of phomenin metabolites produced by this fungus.

## Materials and methods

### Isolation and identification of the fungal isolate 079cE1.2

The fungus was isolated in 2010 from driftwood in Ytre Syltevika on the Varanger Peninsula, at the Barents Sea in the northeastern part of Finnmark County, Norway. A detailed description of the isolation process is provided in the referenced study^20^. The fungal culture was preserved as mycelium on pieces of agar in 20% glycerol solution at − 80 °C and revived for this study on Petri dishes containing low-nutrient malt extract medium prepared in artificial sea water (D2MAA), which consisted of 4 g L^−1^ malt extract (Sigma-Aldrich), 40 g L^−1^ Instant Ocean sea salts (Aquarium Systems), 15 g L^−1^ agar (A1296, Sigma-Aldrich), and Milli-Q^®^ H_2_O. The pH of the medium was 7.35. This revived culture was used as a starting point for the present morphological and chemical investigations. Fresh mycelium was preserved in the in-house glycerol stock and also deposited at the CBS culture collection hosted at the Westerdijk Fungal Biodiversity Institute in the Netherlands.

Morphological investigations of the isolated fungal cultures were conducted using an Olympus SZX16 dissecting microscope and a BX43 light microscope, and photographs were taken using an Olympus DP23 camera. The DNA was amplified in-house using a direct PCR method where a pure fungal colony was swabbed for living cells using a sterile disposable inoculation loop, and the cells were inoculated into 100 µL of autoclaved Milli-Q H_2_O in a 1.8 mL Eppendorf tube. The sample was stored at −20 °C until PCR amplification. DNA was also extracted, amplified, and sequenced in Guelph, Canada, in the facilities of Barcode of Life Systems (BOLD)^21^. The primers used for PCR amplifications of the sequenced genes 28S, ITS, 18S, tef1, and btub are detailed in Supplementary Table S1. ITS, tef1, and btub gene amplicons were cleaned, and a sequencing PCR was run with the same primers used for DNA amplification. The resulting forward and reverse amplicons were cleaned and sent for sequencing at the University Hospital in Northern Norway. The chromatograms of the resulting sequences were checked manually, and consensus sequences were formed using Geneious Prime (www.geneious.com). The consensus sequences generated for this study are deposited in GenBank under accession numbers PV682574–PV682576 and PV686109–PV686110.

The phylogenetic analyses were run using a maximum likelihood and a Bayesian inference method in Geneious Prime. Individual gene sequences were subjected to BLAST analyses against the non-redundant (nr) database in GenBank. Sequences with high similarity BLAST matches were downloaded. MAFFT v.7.490 alignments for each locus were performed using the sequences derived from the isolate 079cE1.2^22^. Alignments were manually adjusted, trimmed at both ends, and concatenated, resulting in a 4410 bp long matrix containing nineteen genetically distinct sequences from twenty *Parafenestella* isolates and the *Paradendryphiella salina* isolate, which was used as an outgroup taxon (Supplementary Table S2). The alignment was divided into partitions based on the five individual genetic loci used and for which individual substitution models were estimated under gamma distribution. For all partitions, a general time-reversible model was used and the maximum likelihood (ML) analysis was executed using 1000 rapid bootstrap (bp) inferences followed by subsequent ML search for best-scoring tree in RAxML^23^. To check the consistency of results, Bayesian analyses were run on the same alignment using MrBayes v. 3.2.6^24^. In these analyses, a general time-reversible (GTR) substitution model and gamma-distributed rate variation with four categories were used. The Markov Chain Monte Carlo (MCMC) analysis was run using a random starting tree with two heated chains, each with a chain length of ten million generations, and the chains were sampled at 2500 generations intervals. The runs reached stationarity as judged by visually inspecting the MCMC trace and with the standard deviation of split frequencies being < 0.01. One million generations were discarded as burn-in and the remaining 3601 trees sampled were used to calculate the posterior probability (PP) support values for the nodes of the 50% majority rule consensus tree.

### Fermentation and extraction of compounds from the 079cE1.2 isolate

Fresh mycelium was cultivated on a Petri dish containing D2MAA medium. A small agar plug with fungal mycelium was transferred into each of 36 culture flasks, each containing 500 mL D2MA (4 g L^−1^ malt extract, 40 g L^−1^ Instant Ocean Sea salts, and Milli-Q^®^ H_2_O) broth medium. The total culture volume of 18 L (36 × 500 mL) was incubated at 20 °C for 35 days under static conditions. After fermentation, the culture media were filtered through cheese cloths (Dansk hjemmeproduktion, Ejstrupholm, Denmark) to obtain mycelium-free broths. The broths were subjected to liquid-liquid extraction two times with ethyl acetate (EtOAc), and the combined EtOAc layer was then evaporated under reduced pressure at 40 °C to obtain a dry extract.

### Screening of the fungal extract for antibacterial activity

The antibacterial activity of the extract from the isolate 079cE1.2 was tested using the broth microdilution method following European Committee for Antimicrobial Susceptibility Testing guidelines of the European Society of Clinical Microbiology and Infectious Diseases (ESMID)^25^ and standard test protocol^26^. The five reference bacterial strains used for initial screening were *Enterococcus faecalis* (ATCC 29122), *Staphylococcus aureus* (ATCC 25923), *Streptococcus agalactiae* (ATCC 12386), *Escherichia coli* (ATCC 25922), and *Pseudomonas aeruginosa* (ATCC 27853). The bacterial strains were grown on blood agar plates at 37 °C for 24 h. Then, the cultures of each bacterium in a growth medium were diluted in fresh medium to achieve an optical density (OD) of 0.5 McFarland (1 × 10^8^ CFU mL^−1^). In this experiment, bacterial cultures were first diluted at a ratio of 1:100 and then further diluted at 1:10 in growth media. The final concentrations of bacterial cells were adjusted to 0.5–3 × 10^5^ for *S. aureus*, *E. coli*, *E. faecalis*, and *S. agalactiae* and 3–7 × 10^4^ CFU mL^−1^ for *P. aeruginosa*. In 96-well plates (Nunclon™ Delta Surface, Thermo Fisher Scientific, Denmark), 50 µL of the bacterial suspension and 50 µL of the extract were added to each well and mixed thoroughly, resulting in a final assay concentration of 250 µg mL^−1^. Gentamycin (Biochrom GmbH, part of Merck Millipore, Germany) was used as a reference (positive control), a growth medium without bacteria was used as a negative control, and bacterial suspension with Milli-Q^®^ water was used as a growth control. After an incubation of 24 h at 37 °C, bacterial growth was assessed by measuring the optical density (OD_600nm_) using a microplate reader (1420 Victor^3TM^ multilabel counter, PerkinElmer^®^, Singapore).

### Prefractionation and bioactivity of the fractionated extract

The extract was fractionated using a Biotage SP4 Flash Chromatography System with a self-packed Biotage SNAP 10 g cartridge column containing Diaion^®^ HP20SS resin as the stationary phase and eluted with a stepwise gradient of H_2_O-MeOH (5:95, 25:75, 50:50, 75:25, 0:100; 6 min per step) followed by MeOH-Acetone (50:50 over 4 min, 0:100 over 10 min) at 12 mL min^−1^ flow rate to afford eight fractions (Fr.1 to Fr.8). These fractions were tested for antibacterial activity towards five pathogenic bacteria at 200 µg mL^−1^.

### UHPLC-HR-MS^E^ analysis and dereplication

A UHPLC-HR-MS^E^ [Acquity I-class ultra-high-performance liquid chromatography system (Waters, Milford, MA, United States) with an Acquity UPLC^®^ BEH C18 column (100 mm × 2.1 mm, 1.7 μm) coupled to a PDA detector and Vion^®^ IMS QTof high-resolution mass spectrometer (Waters, Milford, MA, USA) with positive ionization and data independent acquisition (MS^E^) mode] was used for high-resolution mass spectrometry to analyse the extract. The system was controlled by UNIFI 1.9.4 Scientific Information System. The gradient run was up to 12 min, increasing from 10% to 90% acetonitrile with 0.1% formic acid in Milli-Q water, with a flow rate of 0.45 mL min^−1^.

### Isolation and structure elucidation of compounds from the fungus 079cE1.2

Fractions Fr.5 and Fr.6 were subjected to prep HPLC-MS (Waters Auto Purification HPLC system consisting of a 600 HPLC pump with a 2767 sample manager, a 2996 PDA detector, and a 3100-mass spectrometer operated by MassLynx version 4.1) to isolate three targeted compounds (**1**‒**3**). After the first purification step, interconversion of the compounds was observed. To evaluate their stability, the compounds **1**–**3** were stored at 5 °C immediately after isolation, and their chemical composition was monitored over time to assess their stability and detect any potential formation of degradation products. The compounds were analyzed on Day 1, Day 2, and Day 5 using UHPLC-HR-MS^E^, which consisted of a UPLC BEH C18 column (100 mm × 2.1 mm, 1.7 μm) with solvent A (0.1% formic acid in water) and solvent B (0.1% formic acid in acetonitrile) as mobile phases, at a flow rate of 0.45 mL min^−1^.

The resulting compounds were subjected to a second purification step. Only two compounds (**2** and **3)** were successfully isolated and obtained as pure compounds. The structures of **2** and **3** were determined using 1D and 2D NMR experiments. NMR spectra were acquired in DMSO-*d*_6_ at 25 °C on a Bruker AVANCE III spectrometer operating at 600 MHz for ^1^H, which was equipped with a cryogenically enhanced TCI probe. Detailed information on methods of isolation and structure elucidation of compounds is given in Supplementary Material pages 8–16.

### Antibacterial activity of the isolated compounds

The antibacterial activity of **2** and **3** was assessed against five pathogenic bacteria using the broth microdilution method. Compounds **2** and **3** were dissolved in 20% DMSO to prepare initial stock solutions with concentrations of 10 mM. These stock solutions were subsequently diluted with Milli-Q^®^ water to prepare 250 µM solutions of each compound. Only **2** was tested in duplicate at a final concentration of 125 µM. To determine the minimum inhibitory concentration (MIC) of the compounds, **2** was tested at concentrations of 50, 100, 150, and 200 µM, while **3** was tested at 125, 150, and 200 µM against *S. agalactiae*. For the preparation of sample solutions, the initial stock solutions were further diluted using Milli-Q^®^ water to obtain concentrations of 1000, 800, 400, 200, 100 µM. From the 1000 µM sample solution, additional dilutions were made to prepare 600 and 300 µM sample solutions. For the MIC assay, only the 100, 200, 300, and 400 µM sample solutions of **2** were used, which were further diluted to achieve final test concentrations of 50, 100, 150, and 200 µM, respectively. Similarly, only the 250, 300, and 400 µM sample solutions of **3** were used for the MIC assay, which were further diluted to achieve final test concentrations of 125, 150, and 200 µM, respectively. The MIC values of the compounds were determined as the lowest concentration that completely inhibited bacterial growth, as assessed by measuring OD. An OD value ≤ 0.05 was considered active.

### Antibiofilm formation activity

The effectiveness of **2** in inhibiting biofilm formation was evaluated against *Staphylococcus epidermidis* (ATCC 35984) at concentrations of 50, 100, and 200 µM. In this assay, bacteria were first cultured on blood agar medium from cryostocks, then transferred to tryptic soy broth (TSB, 22092, Sigma-Aldrich) and incubated for about 20 h at 37 °C with shaking at 100 rpm. The cultures were subsequently diluted 1:100 in fresh TSB supplemented with 1% glucose. A 96-well microtiter plate was used, with each well containing 50 µl of the diluted bacterial suspension. An equal volume of sample was added to make a final volume of 100 µl per well. As controls, a non-biofilm-producing *S. haemolyticus* strain was used as a negative control, while an untreated *S. epidermidis* culture was used as a growth control. The bacterial suspension was removed and washed with water, followed by heat fixation of the biofilm at 65 °C. The biofilm was stained with 70 µl of 0.1% crystal violet for 5 min. The crystal violet solution was removed, and the wells were washed with water before the plates were heated. Bound crystal violet was dissolved in 70 µl of 70% ethanol by shaking for 5 min, and OD was measured at 600 nm using a 1420 multilabel counter VICTOR^3TM^ reader.

## Results and discussion

### Phylogenetic placement and morphological description of the 079cE1.2 isolate

**Fig. 1:**
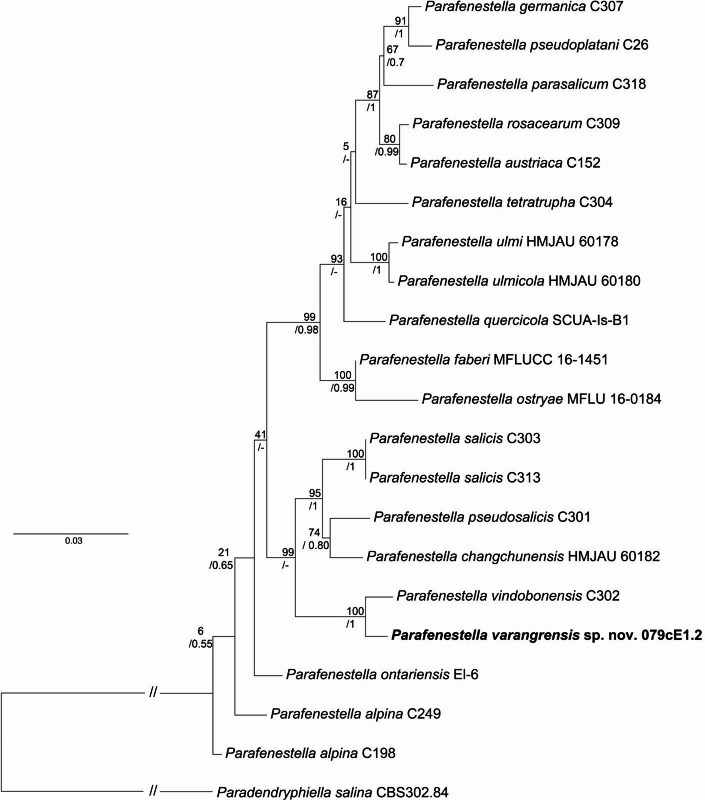
The best-scoring maximum-likelihood tree showing the phylogenetic placement of the isolate 079cE1.2 within the genus *Parafenestella*. The Bayesian analysis produced a similar tree (Supplementary Figure S1) with differences in the topology around certain terminal nodes and the node splitting into the *P. ontariensis* branch and the two more derived clades shown as polytomy. The node support is given as BP and PP values (- when missing), *Paradendryphiella* *salina* was used as an outgroup taxon and the scale bar shows the rate of nucleotide substitutions per site.

The isolate 079cE1.2 was tentatively identified as a *Parafenestella* species based on BLAST searches using the ITS sequence. Based on significant differences of the ITS sequence compared to available reference sequences in GenBank, as well as the previously undocumented ecology and geographical origin of the isolate compared to known *Parafenestella* species, the fungus was sequenced for additional marker genes. These sequences were used in phylogenetic analyses, which showed that the isolate represented an undescribed species consistently clustering with *P. vindobonensis* with statistical node support in all analyses conducted (Fig. 1). These two species form a sister group to a clade containing *P. salicis*, *P. pseudosalicis*, and *P. changchunensis*. All used marker genes gave *P. vindobonensis* as top BLAST match in the NCBI’s database, except for 18S where no sequences for *P. vindobonensis* were available. It is known that 18S, ITS, and 28S markers have low resolution in identifying species within *Cucurbitaceae* family^2^, and this was also the case for the *Parafenestella* isolate 079cE1.2. The ITS and 28 S sequences were 98.9% and 99.9% similar to the *P. vindobonensis* isolate C302, respectively. However, the protein-coding gene beta-tubulin (btub) that is known to give a strong signal within *Cucurbitaceae* was only 96.8% similar between *P. vindobonensis* and our isolate. Additionally, the geographical location and ecology of *P. vindobonensis* are significantly different from *P. varangrensis*. The first mentioned was isolated from a water park (Wasserpark) in Vienna, Austria from *Salix babylonica*, whereas *P. varangrensis* was sourced from spruce (*Picea*) in the Arctic marine environment.

### Taxonomy

**Fig. 2:**
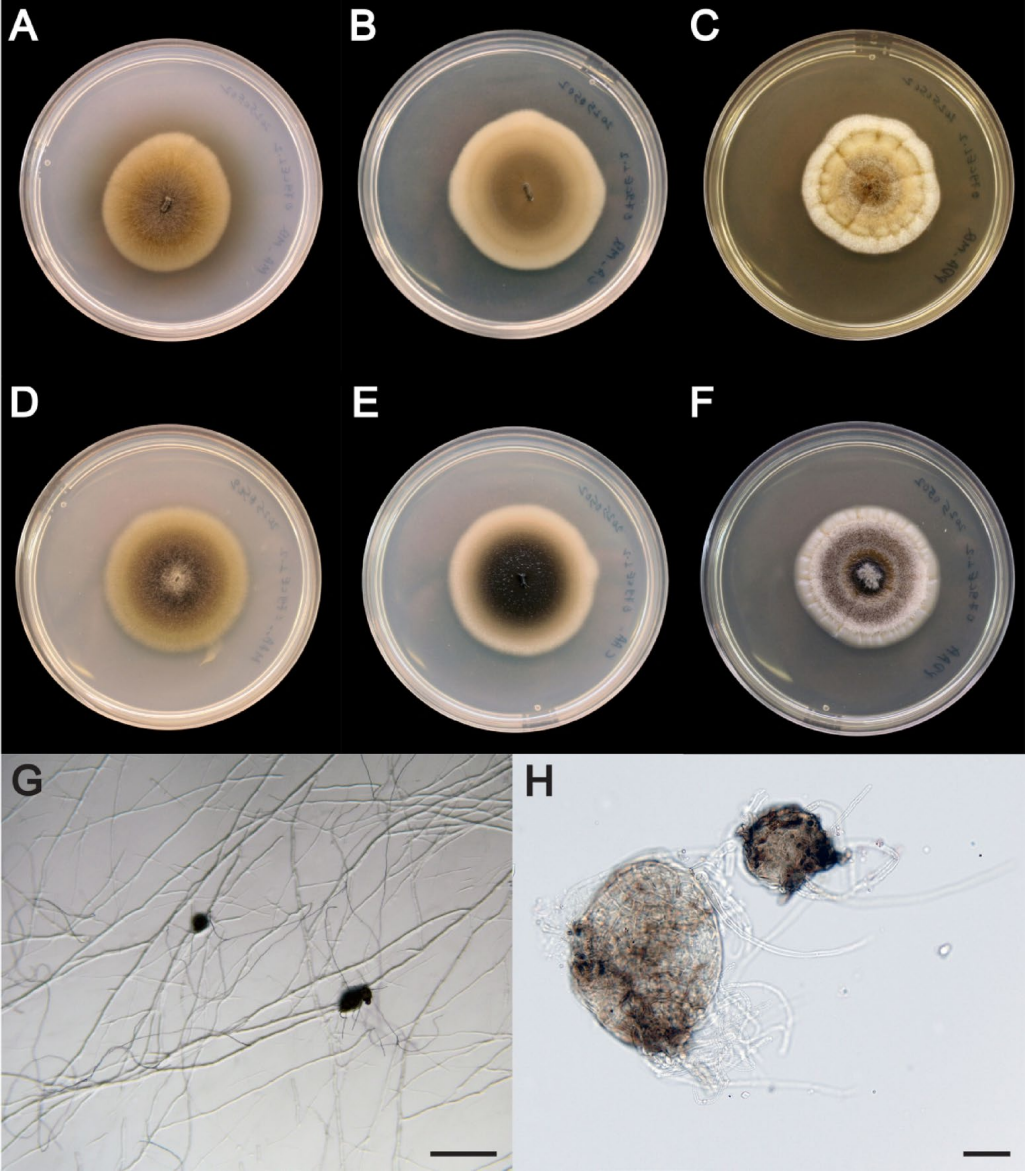
*P. varangrensis*. (**A**‒**F**) colonies of pure culture isolate on different media after 31 days of incubation at 16 °C. (**A**) malt extract agar prepared in Milli-Q® H_2_O. (**B**) corn meal agar in Milli-Q® H_2_O. (**C**) potato-dextrose agar in Milli-Q® H_2_O. (**D**) malt extract agar prepared with artificial seawater (AF). (**E**) corn meal agar prepared in AF, (**F**) potato-dextrose agar prepared in AF. (**G**) Sterile pycnidia-like structures developing in culture. Stereo microscope picture, scalebar = 200 μm. (**H**) Light microscope picture of the structures, scalebar = 20 μm.

*Parafenestella varangrensis* Rämä, sp. nov., Fig. 2, Mycobank MB859231.

*Etymology*: *varangrensis*, referring to the Varanger peninsula (“Varangr” in Old Norse language) where the fungus was isolated.

Cultures consisting of sterile mycelium. On corn meal agar (CA) medium, the fungus produces pycnidia-like structures that are round to ellipsoid in shape and 150–300 μm in diameter (Fig. 2). These remain sterile and no conidiogenous cells or conidia can be seen.

Culture characteristics: Colonies on malt extract agar (MA) medium: 4.0 cm in diameter after 31 days at 16 °C, flat, shape regular, margin entire, greyish white, greyish brown with olivaceus tinge in the centre, moderate aerial hyphae, reverse surface grey, dark greyish brown in the middle, olivaceous pigments secreted in the medium, odour absent; on MA prepared with artificial seawater (Instant Ocean, Aquarium systems, UK): 4.1 cm in diameter after 31 days at 16 °C, flat, shape regular, margin entire, greyish white, olive brown towards the centre, centre greyish brown, moderate aerial hyphae, reverse surface concolorous with top, no pigments secreted to the medium, odour absent.

Colonies on CA: 4.2 cm in diameter after 31 days at 16 °C, flat, shape regular, margin entire, whitish, light brown in the middle, aerial hyphae absent, reverse surface grey, dark brownish grey in the middle, no pigments secreted in the medium, odour absent; on CA prepared with artificial seawater: 4.2 cm in diameter after 31 days at 16 °C, flat, shape regular, margin entire, greyish white, dark brown in the middle with olivaceous tinge, shiny, aerial hyphae absent, reverse surface concolorous with the top, no pigments secreted to the medium, odour absent.

Colonies on potato dextrose agar (PDA) medium: 4.2 cm in diameter after 31 days at 16 °C, umbonate, shape regular, margin clearly defined, radially striate with lobate edge, greyish white aerial hyphae dense creating a hairy surface, colony surface yellowish white in colour, olivaceous, yellowish zones towards the centre that is yellowish brown, reverse surface creamy in colour, sharp greenish grey zone, yellowish in the centre, no pigments secreted in the medium, odour absent; on PDA prepared with artificial seawater: 4.2 in diameter after 31 days at 16 °C, umbonate, shape regular, margin clearly defined, radially striate with lobate edge, aerial hyphae dense creating a hairy surface, colony surface edge greyish white with yellowish hairless inner part, followed by hairy zones of greyish and dark grey, hairless zone of yellowish grey and brown, centre hairy and grey, reverse surface yellowish whited, followed by narrow dark grey zone, grey zone and dark grey in the middle, no pigments secreted to the medium, odour absent.

Unfortunately, the isolate remained sterile, not developing diagnostic asexual or sexual traits, despite being cultured on six different media for several months in the laboratory. This impeded the morphological comparison to other *Parafenestella* species. However, in addition to the unique substrate and geographical origin of the fungus within the genus *Parafenestella*, we have evidence from the multilocus phylogenetic analysis to show that our isolate represents a previously undescribed species.

*Typus*: Norway, Finnmark County, Båtsfjord Municipality, Ytre Syltevika, 70°32’55’' N 30°24’21’' E, the fungus was isolated from a stranded driftwood log of spruce (*Picea*) that was located in the upper splash zone on a stony shore, the log was loose, had average diameter of 14 cm and length of 5.1 m, was hard (decay class 1 at a scale from 1 to 5) and had holes made by marine boring invertebrates, 8 September 2010, T. Rämä, 079cE1.2; holotype TROM-F-26890 freeze-dried culture on malt extract agar medium, stored at Tromsø university museum fungarium; ex-holotype culture CBS153929; 18S, ITS, 28S as well as tef1 and tub sequences in GenBank PV 682574–PV682576 and PV686109–PV686110, respectively; MycoBank MB859231.

### UHPLC-HR-MS^E^ analysis, dereplication, and bioactivity screening

**Fig. 3:**
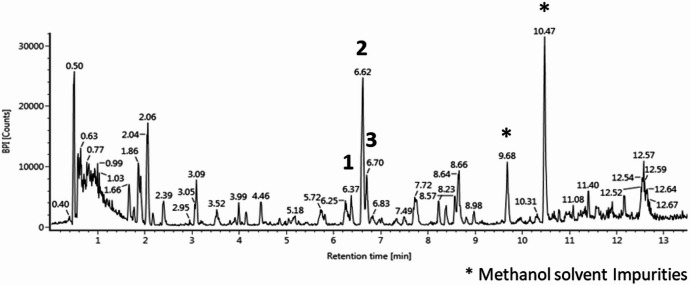
UHPLC-HR-MS^E^ base peak ion chromatogram of the extract of *P. varangrensis*.

A total of 1.62 g of crude extract was obtained from an 18 L culture of *P. varangrensis*. The extract was analysed for its chemical constituents using UHPLC-HR-MS^E^, revealing the presence of three compounds with identical masses and elemental compositions (Fig. 3). Dereplication of these compounds using the ChemSpider, and Natural Product Atlas databases indicated phomenin (C_14_H_18_O_3_) as one of the potential hits. However, the exact structures of the compounds could not be identified because phomenins exist in different structural variations, known as isomers. The crude extract was screened for antibacterial activity against five pathogenic bacterial strains. The extract showed activity against two Gram-positive pathogenic bacteria, *E. faecalis* and *S*. *agalactiae* (Supplementary Figure S2).

### Bioactivity of fractionated extract and isolation of compounds

**Fig. 4:**
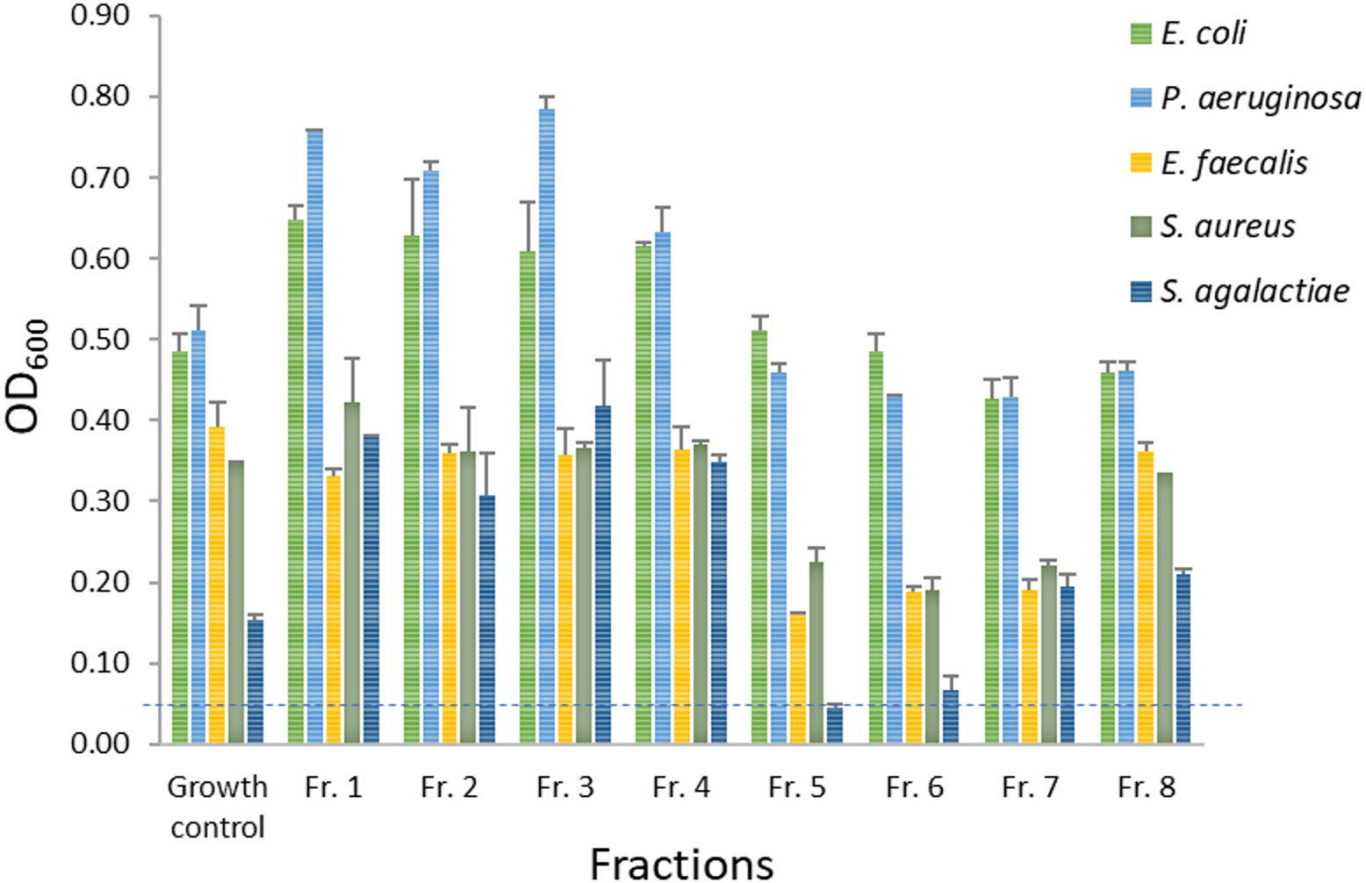
Antibacterial activity of eight flash fractions (Fr.1−Fr.8) against five pathogenic bacteria. Activity was assessed by measuring OD at 600 nm. Samples with an OD below 0.05 were classified as active.

The crude extract was fractionated into eight fractions using flash chromatography, and the fractions were tested for antibacterial activity at 200 µg mL^−1^. Fraction 5 (Fr.5) was active against *S. agalactiae*, while fraction 6 (Fr.6) also exhibited some inhibitory effect (∼73% inhibition) on the growth of *S. agalactiae*. The other fractions showed no activity against any of the tested bacteria (Fig. 4). All flash fractions were analysed for their chemical constituents using UHPLC-HR-MS^E^. The analysis revealed that Fr.5, and Fr.6 shared similar chemical profiles, each containing the same major compounds. These fractions were combined, subjected to prep HPLC-MS, and yielded compounds **1**, **2**, and **3** (Supplementary Figure S4).

After the first purification step, we found that **1**, **2**, and **3** were isomerized into one another when they were analyzed using UHPLC-HR-MS^E^ (Supplementary Figure S5). This conversion might be due to the exposure to elevated temperatures while drying the isolated compounds, which involved evaporation under reduced pressure at 40 °C. To confirm the effect of elevated temperature on the interconversion of isomers, the compounds were dried at lower temperatures, and their stability was monitored using UHPLC-HR-MS^E^. The stability of the compounds was evaluated on the day of isolation (Day 1), Day 2, and Day 5. All isomers remained stable when stored at 5 °C (Supplementary Figures S6–8). Therefore, all these compounds were further purified (second purification step) with minimal light exposure, concentrated at lower temperatures (30 °C) for a few minutes using a rotary evaporator, and finally freeze-dried. Compounds **2** and **3** were successfully obtained as pure compounds after the second purification step (Supplementary Figure S9). However, isolating **1**, free from other isomers, was unsuccessful, and the small amount recovered after the second purification step was insufficient for further purification. Compound **1** appeared to convert readily to **2**, likely due to *Z*→*E* isomerization, even during short exposure to 30 °C while drying and subsequent handling at room temperature. Therefore, **1** was not considered for further studies.

### Identification of compounds

**Fig. 5:**
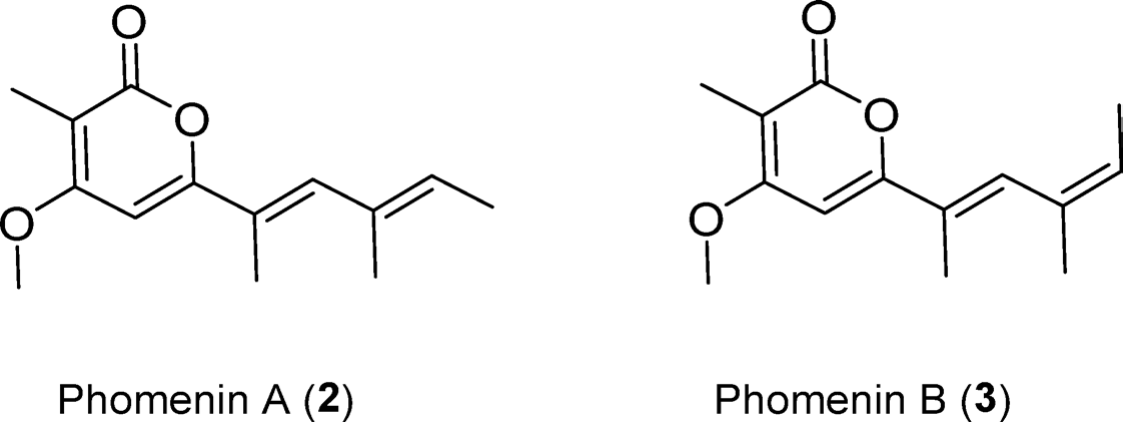
Structures of **2** and **3** isolated from the extract of *P. varangrensis*.

Two pure compounds, **2** and **3**, isolated from *P. varangrensis* were obtained as light reddish-brown solids. The structures of these two compounds were elucidated through analysing 1D and 2D NMR spectra. Detailed assignments of NMR signals and the elucidation of structures are provided in the Supplementary Material pages 14–16. Compounds **2** and **3** were identified as phomenin A and phomenin B, respectively (Fig. 5). Both compounds belong to the propionate α-pyrone-derived polyketide class of secondary metabolites. Phomenin B is the geometric isomer of phomenin A at the ∆^9^-double bond. These compounds have previously been isolated from phytopathogenic fungal species such as *Phoma tracheiphila*, *Leptosphaeria maculans*, and *Alternaria infectoria* that also are placed in the order *Pleosporales*. However, they have not previously been isolated from *Parafenestella* spp. or any marine-derived fungus. These compounds have been reported under different names (phomapyrone A and B) in earlier studies^6^.

We detected another isomer of phomenin (**1**) (Supplementary Figure S3) that we could not isolate in sufficient quantity for further characterization and activity testing. Compound **1** had a low yield after a second purification step and existed as a mixture of isomers, so its structure could not be elucidated. Elemental composition and MS spectra of **1** match those of compounds **2** and **3**. We therefore speculate that **1** could be another isomer of phomenin with either 7*Z*, 9*Z* or 7*Z*, 9*E* configurations, unlike **2** (7*E*, 9*E*) and **3** (7*E*, 9*Z*). The speculated structure of **1** still needs confirmation through NMR data. Compound **1** was found in low abundance and was observed to be less stable than **2** and **3**. Based on the speculated structure, it likely contains at least a *Z* configuration at the Δ^7^-double bond, or more likely, at both the Δ^7^- and Δ^9^-double bonds. This is because *Z*-isomers are generally less thermodynamically stable than *E*-isomers^27–29^. Additionally, the activation energy for the *Z*→*E* transformation is smaller than that for the reverse *E*→*Z* transformation^30^. These reasons explain why **2** was more stable than **1** and **3**, with **1** being the least stable. The higher stability of **2** could be due to its 7*E*, 9*E* configuration in the alkene side chain, which reduces steric hindrance and favours a more thermodynamically stable arrangement. Moreover, when the compounds were exposed to elevated temperatures during drying, thermal energy could overcome the activation energy required to temporarily weaken the π-bond. This allowed rotation around the double bond, potentially facilitating *E*/*Z* isomerization. Previous studies on the synthesis of pyrone-derived compounds have demonstrated that polyene or double-bond (*E*/*Z*) isomerization can be induced through thermal, photochemical, or other reaction means^31^. Similarly, the synthesis of polypropionate-derived γ-pyrone compounds has shown that the yield of *E*-isomers is usually higher than that of *Z*-isomers, suggesting that *E*-isomers are thermodynamically more stable^32^. The observed differences in stability among compounds **1**–**3** can therefore be attributed to their *E*/*Z* configurations and the energy required for isomerization under thermal conditions. We therefore hypothesize that **1** can only be isolated and handled at low temperature and with minimal light exposure.

### Evaluation of antibacterial activity and biofilm inhibition of compounds **2** and **3**

**Fig. 6:**
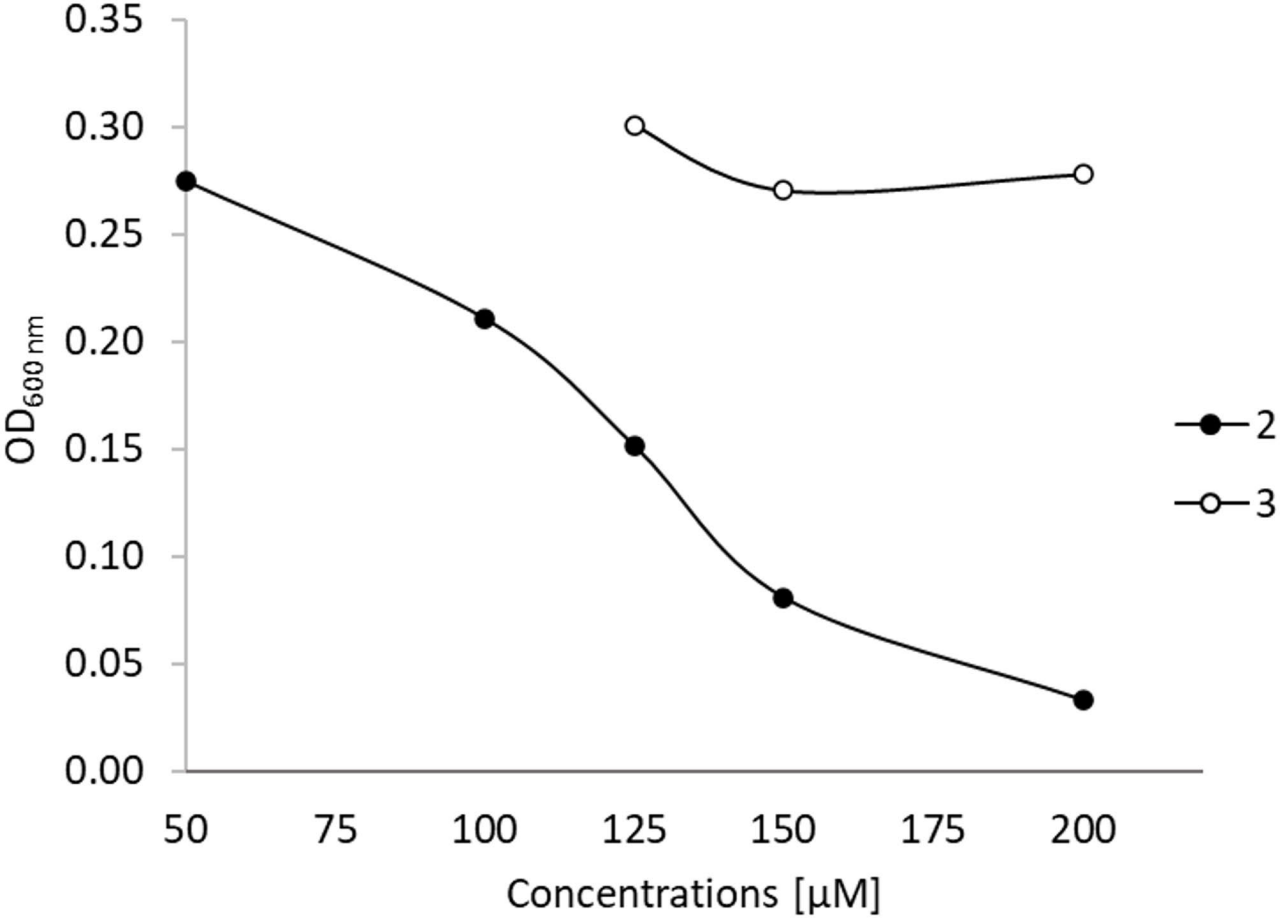
Antibacterial activity of **2** and **3** against *S. agalactiae*. Compound **2** was tested at concentrations of 50, 100, 150, and 200 µM, while **3** was tested at concentrations of 125, 150, and 200 µM. Bacterial growth was measured at OD600 nm, and an OD value ≤ 0.05 was considered active.

Because of the low yields of the pure compounds, only **2** could be profiled for antibacterial activity against *E. coli*, *P. aeruginosa*, *E. faecalis*, and *S. aureus*. Among the tested bacterial strains, the growth of *S. agalactiae* was partially inhibited (~ 38% inhibition) by **2** at a concentration of 125 µM (Supplementary Figure S34). A much higher concentration of **2** was needed to completely inhibit the growth of *S. agalactiae*. To compare the antibacterial activity of **2** and **3** against *S. agalactiae*, serial dilutions of the compounds were added to *S. agalactiae* cultures in 96-well plates, and the growth of the bacterium was determined. Among the two test compounds, only **2** exhibited dose-dependent growth inhibitory activity against *S. agalactiae*, with an MIC value of 173 µM (Fig. 6, Supplementary Figures S35 and S36). This highlights that, despite their minor structural differences, compounds **2** and **3** exhibited significantly different antibacterial activities.

Compound **2** was further evaluated for inhibition of biofilm formation by *S. epidermidis* at concentrations of 50, 100, and 200 µM (Supplementary Figure S37). Biofilm formation was weakly inhibited at 200 µM, but the effect was not significant for the compound to be considered active. Compound **3** was not tested in this assay due to limited availability. Further studies should explore the potential of *P. varangrensis* to produce other isomers of phomenin, including phomenin B. Optimizing cultural conditions to enhance the production of these minor isomers, combined with their detailed characterization, could provide valuable insights into their antibacterial and biofilm inhibition properties.

In this study, we present the first report on the antibacterial effect of phomenin A. Moreover, phomenins A and B have been reported to exhibit cytotoxic and phytotoxic activities^5,8^. A mixture of phomenins A and B exerted a cytotoxic effect against human lung fibroblast (MRC-5) cells in the Neutral Red uptake assay^8^. Further studies on the cytotoxic activity of pure phomenins against MRC-5 and other cell lines need to be explored. These compounds may also possess insecticidal properties, as they have previously demonstrated activity in the brine shrimp bioassay^5^. Phomenins are significant due to their diverse biological activities and could play a role in plant-pathogen interactions, making them important for agricultural research aimed at managing plant diseases. Additionally, the presence of the α-pyrone pharmacophore in their structures, along with their bioactivity profiles, highlights their potential as scaffolds for structural optimization, which could lead to the development of potential drug candidates^10,33^. While this study provides valuable insights into the antibacterial and biofilm inhibition properties of phomenins, there are some limitations. Due to the low yield of the compounds, we were unable to explore other bioactivities. Furthermore, the low potency of **2** for antibacterial activity limits its immediate application. Further studies are needed to explore the full potential of phomenins.

## Conclusions

In summary, *Parafenestella varangrensis*, a driftwood-associated fungus, was described and evaluated for its production of bioactive secondary metabolites. This resulted in the isolation and characterization of two known α-pyrone-derived polyketides, phomenins A (**2**) and B (**3**). Only phomenin A inhibited the growth of one of the tested bacteria, *S. agalactiae*. Additional studies to isolate more and evaluate the biofilm inhibition potential of phomenin B can be performed. Moreover, the production of other minor phomenin isomers, particularly **1**, can be explored and their structures elucidated.

## Supplementary Information

Below is the link to the electronic supplementary material.

Supplementary Material 1

## Data Availability

The sequencing data generated for this study are available in the NCBI GenBank and European Nucleotide Archive databases under the accession numbers PV682574–PV682576 and PV686109–PV686110. *P. varangrensis* culture is deposited at the CBS culture collection at the Westerdijk Fungal Biodiversity Institute in the Netherlands (https://wi.knaw.nl/fungal_table) under accession number CBS 153929. The holotype of the fungus TROM-F-26890, a freeze-dried culture, is deposited at the Arctic University Museum of Norway Fungarium and voucher information can be accessed at the Global Diversity Information Facility (https://www.gbif.org/occurrence/5203505382). Other data are available in the article and its supplementary material.
