# Supplementary material for: Parafenestella varangrensis sp. nov., a phomenin producing fungus from the Arctic
Source: Sci Rep. 2025 Dec 23;16:3221. doi: 10.1038/s41598-025-33070-y (PMC12830994; doi:10.1038/s41598-025-33070-y)
Supplement: Supplementary file 1 — Supplementary Material 1 [file 41598_2025_33070_MOESM1_ESM.docx]

SUPPLEMENTARY INFORMATION

*Parafenestella varangrensis* sp. nov., a phomenin producing strain from the Arctic

Sailesh Maharjan^1,^*, Julie Marie Lesjø^2^, Johan Isaksson^2, 3^, Kine Østnes Hansen^2^, Jeanette Hammer Andersen^1^, Espen Holst Hansen^1^, Teppo Rämä^1^

^1^Marbio, Norwegian College of Fishery Science (NFH), Faculty of Biosciences, Fisheries, and Economics, UiT-The Arctic University of Norway, Tromsø 9037, Norway

^2^Department of Pharmacy (IFA), Faculty of Health Sciences, UiT-The Arctic University of Norway, Tromsø 9037, Norway

^3^Department of Chemistry (IK), Faculty of Science and Technology, UiT-The Arctic University of Norway, Tromsø 9037, Norway

*****Corresponding author:

Email: [sailesh.maharjan@uit.no](mailto:sailesh.maharjan@uit.no); Phone: +4796804941

KEYWORDS

Antibacterial α-pyrone, polyketide, phomenin, *Parafenestella*, *varangrensis*, marine-derived fungus, Arctic

Table of Contents

[Isolation of compounds from the fungus 079cE1.2 8](#_Toc206352658)

[First purification step 8](#_Toc206352659)

[Monitoring the stability of the compounds 8](#_Toc206352660)

[Second purification step 8](#_Toc206352661)

[Structure elucidation of isolated compounds 14](#_Toc206352662)

[Phomenin A (**2**) 16](#_Toc206352663)

[Bioactivity screening 27](#_Toc206352664)

[Determination of minimum inhibitory concentration (MIC) value 28](#_Toc206352665)

[References 29](#_Toc206352666)

**List of tables**

[Table S 1. The primers used for PCR amplifications 4](#_Toc206493279)

[Table S 2. GenBank accession numbers of sequences used to build the phylogenetic tree. Sequences in bold were created for this study. 5](#_Toc206493280)

[Table S 3. ^1^H (600 MHz) and ^13^C (151 MHz) NMR data of 2 and 3 in DMSO-*d_6_*. 16](#_Toc206493281)

**List of figures**

[Figure S 1. Phylogenetic tree from Bayesian analysis with MrBayes, showing the placement of the isolate 079cE1.2. 6](#_Toc206425995)

[Figure S 2. Initial screening of antibacterial activity of the fungal extract towards five pathogenic bacteria. 7](#_Toc206425996)

[Figure S 3. UHPLC-HRMS base peak ion chromatogram of fractions 5 (Fr.5) and 6 (Fr.6) showing the presence of compounds 1, 2, and 3. 7](#_Toc206425997)

[Figure S 4. Preparative HPLC-MS base peak ion chromatogram of Fr.5. First round purification of 1, 2, and 3. 9](#_Toc206425998)

[Figure S 5. UHPLC-HR-MS base peak ion chromatograms of isolated compounds 1, 2, and 3 to assess the stability of the compounds. (a) Chromatogram of 1 shows the detection of all three compounds. (b) Chromatogram of 2 shows the presence of all three compounds. (c) Chromatogram of 3 shows the presence of all three compounds. It suggests that these compounds are unstable and interconvertible with each other when exposed to elevated temperature (40 °C). 9](#_Toc206425999)

[Figure S 6. UHPLC-HR-MS base peak ion chromatogram showing the stability of 1 on Day 1 (a), Day 2 (b), and Day 5 (c) when stored at a low temperature (5 °C). 10](#_Toc206426000)

[Figure S 7. UHPLC-HR-MS base peak ion chromatogram showing the stability of 2 on Day 1 (a), Day 2 (b), and Day 5 (c) when stored at a low temperature (5 °C). 10](#_Toc206426001)

[Figure S 8. UHPLC-HRMS base peak ion chromatogram showing the stability of 3 Day 1 (a), Day 2 (b), and Day 5 (c) when stored at a low temperature (5 °C). 11](#_Toc206426002)

[Figure S 9. Preparative HPLC-MS base peak ion chromatogram of 1 (Second round purification). 11](#_Toc206426003)

[Figure S 10. Preparative HPLC-MS base peak ion chromatogram of 2 (Second round purification). 12](#_Toc206426004)

[Figure S 11. Preparative HPLC-MS base peak ion chromatogram of 3 (Second round purification). 12](#_Toc206426005)

[Figure S 12. HRESIMS of 1 at (a) low collision energy (b) high collision energy in ESI+ mode. 13](#_Toc206426006)

[Figure S 13. Key HMBC (blue arrow) and ROESY (red arrow) correlations of 2 and 3. 15](#_Toc206426007)

[Figure S 14. HRESIMS of 2 at (a) low collision energy (b) high collision energy in ESI+ mode. 17](#_Toc206426008)

[Figure S 15. UV-Vis spectrum of 2. 17](#_Toc206426009)

[Figure S 16. ^1^H NMR spectrum (600 MHz, DMSO-*d_6_*) of 2. 18](#_Toc206426010)

[Figure S 17. ^13^C NMR spectrum (151 MHz, DMSO-*d_6_*) of 2. 18](#_Toc206426011)

[Figure S 18. HSQC spectrum of 2. 19](#_Toc206426012)

[Figure S 19. HMBC spectrum of 2. 19](#_Toc206426013)

[Figure S 20. ^1^H-^1^H COSY spectrum of 2. 20](#_Toc206426014)

[Figure S 21. ROESY spectrum of 2. 20](#_Toc206426015)

[Figure S 22. HRESIMS of 3 at (a) low collision energy (b) high collision energy in ESI+ mode. 21](#_Toc206426016)

[Figure S 23. UV-Vis spectrum of 3. 21](#_Toc206426017)

[Figure S 24. ^1^H NMR of 3 (after the first round of purification). 22](#_Toc206426018)

[Figure S 25. HSQC of 3 (after the first round of purification). 22](#_Toc206426019)

[Figure S 26. HSQC + HMBC of 3 (after the first round of purification). 23](#_Toc206426020)

[Figure S 27. HMBC of 3 (after the first round of purification). 23](#_Toc206426021)

[Figure S 28. COSY of 3 (after the first round of purification). 24](#_Toc206426022)

[Figure S 29. ROESY of 3 (after the first round of purification). 24](#_Toc206426023)

[Figure S 30. ^1^H NMR of 3 (after the second round of purification). 25](#_Toc206426024)

[Figure S 31. ^13^C NMR of 3 (after the second round of purification). 25](#_Toc206426025)

[Figure S 32. ROESY of 3 (after the second round of purification). 26](#_Toc206426026)

[Figure S 33. ROESY (Reduced t1 noise) of 3 (after the second round of purification). 26](#_Toc206426027)

[Figure S 34. Screening of the antibacterial activity of 2 towards five pathogenic bacteria at 125 µM. 27](#_Toc206426028)

[Figure S 35. Antibacterial activity of 2, 3, and gentamycin against *S. agalactiae*. Compound 2 was tested at concentrations of 50, 100, 150, and 200 μM, while 3 was tested at concentrations of 125, 150, and 200 µM. Gentamycin was used as a reference (positive control) and tested at a concentration range of 0.015–32 μM. Bacterial growth was measured at OD600 nm, and an OD value ≤ 0.05 was considered active. 27](#_Toc206426029)

[Figure S 36. Sigmoidal concentration-response curve of 2 showing the MIC value against *S. agalactiae*. 28](#_Toc206426030)

[Figure S 37. Inhibition of biofilm formation of *S. epidermidis* by 2. 29](#_Toc206426031)

Table S 1. The primers used for PCR amplifications

| **Primer pair** | **Primer sequence** | **References** |
| --- | --- | --- |
| ITS5  ITS4 (ITS) | 5’-GGAAGTAAAAGTCGTAACAAGG-3’  5’-TCCTCCGCTTATTGATATGC-3’ | (White et al. 1990) |
| NS1  NS4 (18S part1)  NSSU897R  NS24 (18S part2) | 5´-GTAGTCATATGCTTGTCTC-3´  5´ CTTCCGTCAATTCCTTTAAG-3´  5´- AGAGGTGAAATTCTTGGA -3´  5´-AAACCTTGTTACGACTTTTA-3´ | (White et al. 1990)    (Gargas & Taylor 1992, Lutzoni et al. 2004) |
| LR0R  LR5 (28S) | 5´-GTACCCGCTGAACTTAAGC -3´   5´-ATCCTGAGGGAAACTTC-3´ | (Rehner & Samuels 1994, Vilgalys & Hester 1990) |
| EF1-983F  EF1-1620R | 5´-GCYCCYGGHCAYCGTGAYTTYAT-3´  5´-GACGTTGAADCCRACRTTGTC-3 | (Rehner & Buckley 2005, Stielow et al. 2015) |
| Btub2Fd  Btub4Rd | 5´-GTBCACCTYCARACCGGYCARTG-3´  5´-CCGAYTGRCCRAARACRAAGTTGTC-3´ | (Woudenberg et al. 2009) |

Table S 2. GenBank accession numbers of sequences used to build the phylogenetic tree. Sequences in bold were created for this study.

| **Species** | **Isolate ID** | **Note** | **18S** | **ITS** | **28S** | **tef1** | **Tub2** |
| --- | --- | --- | --- | --- | --- | --- | --- |
| *Paradendryphiella salina* | CBS 302.84 | Outgroup | [KC584583](https://www.ncbi.nlm.nih.gov/nuccore/KC584583.1) | [MH873443](https://www.ncbi.nlm.nih.gov/nuccore/MH873443.1) | [KC584325](https://www.ncbi.nlm.nih.gov/nuccore/KC584325.1) | [JQ672390](https://www.ncbi.nlm.nih.gov/nuccore/JQ672390.1) | [JQ671942](https://www.ncbi.nlm.nih.gov/nuccore/JQ671942) |
| *Parafenestella pseudosalicis* | C301 | ex-holotype culture | na (partial) | [MK356307](https://www.ncbi.nlm.nih.gov/nuccore/MK356307.1) | na (partial) | [MK357579](https://www.ncbi.nlm.nih.gov/nuccore/MK357579.1) | [MK357620](https://www.ncbi.nlm.nih.gov/nuccore/MK357620.1) |
| *Parafenestella salicis* | C303 |  | na (partial) | [MK356316](https://www.ncbi.nlm.nih.gov/nuccore/MK356316.1) | na (partial) | [MK357588](https://www.ncbi.nlm.nih.gov/nuccore/MK357588.1) | [MK357628](https://www.ncbi.nlm.nih.gov/nuccore/MK357628.1) |
| *Parafenestella salicis* | C313 | ex-neotype culture of Thyridium salicis | na (partial) | [MK356317](https://www.ncbi.nlm.nih.gov/nuccore/MK356317.1) | na (partial) | [MK357589](https://www.ncbi.nlm.nih.gov/nuccore/MK357589.1) | [MK357629](https://www.ncbi.nlm.nih.gov/nuccore/MK357629.1) |
| *Parafenestella vindobonensis* | C302 | ex-holotype culture | na (partial) | [MK356320](https://www.ncbi.nlm.nih.gov/nuccore/MK356320.1) | na (partial) | [MK357592](https://www.ncbi.nlm.nih.gov/nuccore/MK357592.1) | [MK357632](https://www.ncbi.nlm.nih.gov/nuccore/MK357632.1) |
| ***Parafenestella sp.*** | **079cE1.2** |  | **PV682574** | **PV682575** | **PV682574** | **PV686109** | **PV686110** |
| *Parafenestella alpina* | C249 |  | na (partial) | [MK356303](https://www.ncbi.nlm.nih.gov/nuccore/MK356303.1) | na (partial) | [MK357575](https://www.ncbi.nlm.nih.gov/nuccore/MK357575.1) | [MK357616](https://www.ncbi.nlm.nih.gov/nuccore/MK357616.1) |
| *Parafenestella alpina* | C198 | ex-holotype culture | na (partial) | [MK356302](https://www.ncbi.nlm.nih.gov/nuccore/MK356302.1) | na (partial) | [MK357574](https://www.ncbi.nlm.nih.gov/nuccore/MK357574.1) | [MK357615](https://www.ncbi.nlm.nih.gov/nuccore/MK357616.1) |
| *Parafenestella changchunensis* | HMJAU 60182 | ex-holotype culture | [OL891808](https://www.ncbi.nlm.nih.gov/nuccore/OL891808.1) | [OL996119](https://www.ncbi.nlm.nih.gov/nuccore/OL996119) | [OL897170](https://www.ncbi.nlm.nih.gov/nuccore/OL897170.1) | [OL944600](https://www.ncbi.nlm.nih.gov/nuccore/OL944600.1) | [OL898719](https://www.ncbi.nlm.nih.gov/nuccore/OL898719.1) |
| *Parafenestella ulmi* | HMJAU 60178 | holotype | [OL891806](https://www.ncbi.nlm.nih.gov/nuccore/OL891806.1) | [NR_191186](https://www.ncbi.nlm.nih.gov/nuccore/NR_191186.1) | [OL897166](https://www.ncbi.nlm.nih.gov/nuccore/OL897166.1) | [OL944596](https://www.ncbi.nlm.nih.gov/nuccore/OL944596.1) | [OL898723](https://www.ncbi.nlm.nih.gov/nuccore/OL898723.1) |
| *Parafenestella ostryae* | MFLU 16-0184 | holotype | [NG_065103](https://www.ncbi.nlm.nih.gov/nuccore/NG_065103.1) | [NR_154365](https://www.ncbi.nlm.nih.gov/nuccore/NR_154365.1) | [KY563075](https://www.ncbi.nlm.nih.gov/nuccore/KY563075.1) | na | na |
| *Parafenestella pseudoplatani* | C26 | holotype/ex-holotype culture | na (partial) | [MF795788](https://www.ncbi.nlm.nih.gov/nuccore/MF795788.1) | na (partial) | [MF795876](https://www.ncbi.nlm.nih.gov/nuccore/MF795876.1) | [MF795914](https://www.ncbi.nlm.nih.gov/nuccore/MF795914.1) |
| *Parafenestella ulmicola* | HMJAU 60180 | holotype | [OL891807](https://www.ncbi.nlm.nih.gov/nuccore/OL891807.1) | [NR_191187](https://www.ncbi.nlm.nih.gov/nuccore/NR_191187.1) | [OL897168](https://www.ncbi.nlm.nih.gov/nuccore/OL897168.1) | [OL944598](https://www.ncbi.nlm.nih.gov/nuccore/OL944598.1) | [OL898724](https://www.ncbi.nlm.nih.gov/nuccore/OL898724.1) |
| *Parafenestella ontariensis* | EI-6 | holotype | na | [NR_184963](https://www.ncbi.nlm.nih.gov/nuccore/NR_184963.1) | [OM286884](https://www.ncbi.nlm.nih.gov/nuccore/OM286884.1) | na | na |
| *Parafenestella germanica* | C307 | holotype | na (partial) | [MK356305](https://www.ncbi.nlm.nih.gov/nuccore/MK356305.1) | na (partial) | [MK357577](https://www.ncbi.nlm.nih.gov/nuccore/MK357577.1) | [MK357618](https://www.ncbi.nlm.nih.gov/nuccore/MK357618.1) |
| *Parafenestella rosacearum* | C309 | holotype | na (partial) | [MK356311](https://www.ncbi.nlm.nih.gov/nuccore/MK356311.1) | na (partial) | MK357583 | na |
| *Parafenestella austriaca* | C152 | holotype | na (partial) | [MK356304](https://www.ncbi.nlm.nih.gov/nuccore/MK356304.1) | na (partial) | [MK357576](https://www.ncbi.nlm.nih.gov/nuccore/MK357576.1) | [MK357617](https://www.ncbi.nlm.nih.gov/nuccore/MK357617.1) |
| *Parafenestella tetratrupha* | C304 | epitype | na (partial) | [MK356319](https://www.ncbi.nlm.nih.gov/nuccore/MK356319.1) | na (partial) | [MK357591](https://www.ncbi.nlm.nih.gov/nuccore/MK357591.1) | [MK357631](https://www.ncbi.nlm.nih.gov/nuccore/MK357631.1) |
| *Parafenesttella parasalicum* | C318 | ex-holotype culture | na (partial) | [MK356306](https://www.ncbi.nlm.nih.gov/nuccore/MK356306.1) | na (partial) | [MK357578](https://www.ncbi.nlm.nih.gov/nuccore/MK357578.1) | [MK357619](https://www.ncbi.nlm.nih.gov/nuccore/MK357619.1) |
| *Parafenestella faberi* | MFLUCC 16-1451 | ex-holotype culture of P. mackenziei (synonym) | [NG_063631](https://www.ncbi.nlm.nih.gov/nuccore/NG_063631.1) | [NR_154364](https://www.ncbi.nlm.nih.gov/nuccore/NR_154364.1) | [KY563074](https://www.ncbi.nlm.nih.gov/nuccore/KY563074.1) | na | na |
| *Parafenestella quercicola* | SCUA-Is-B1 | ex-holotype culture | na | [OR440609](https://www.ncbi.nlm.nih.gov/nuccore/OR440609) | na | [OR450812](https://www.ncbi.nlm.nih.gov/nuccore/OR450812) | [OR450818](https://www.ncbi.nlm.nih.gov/nuccore/OR450818) |


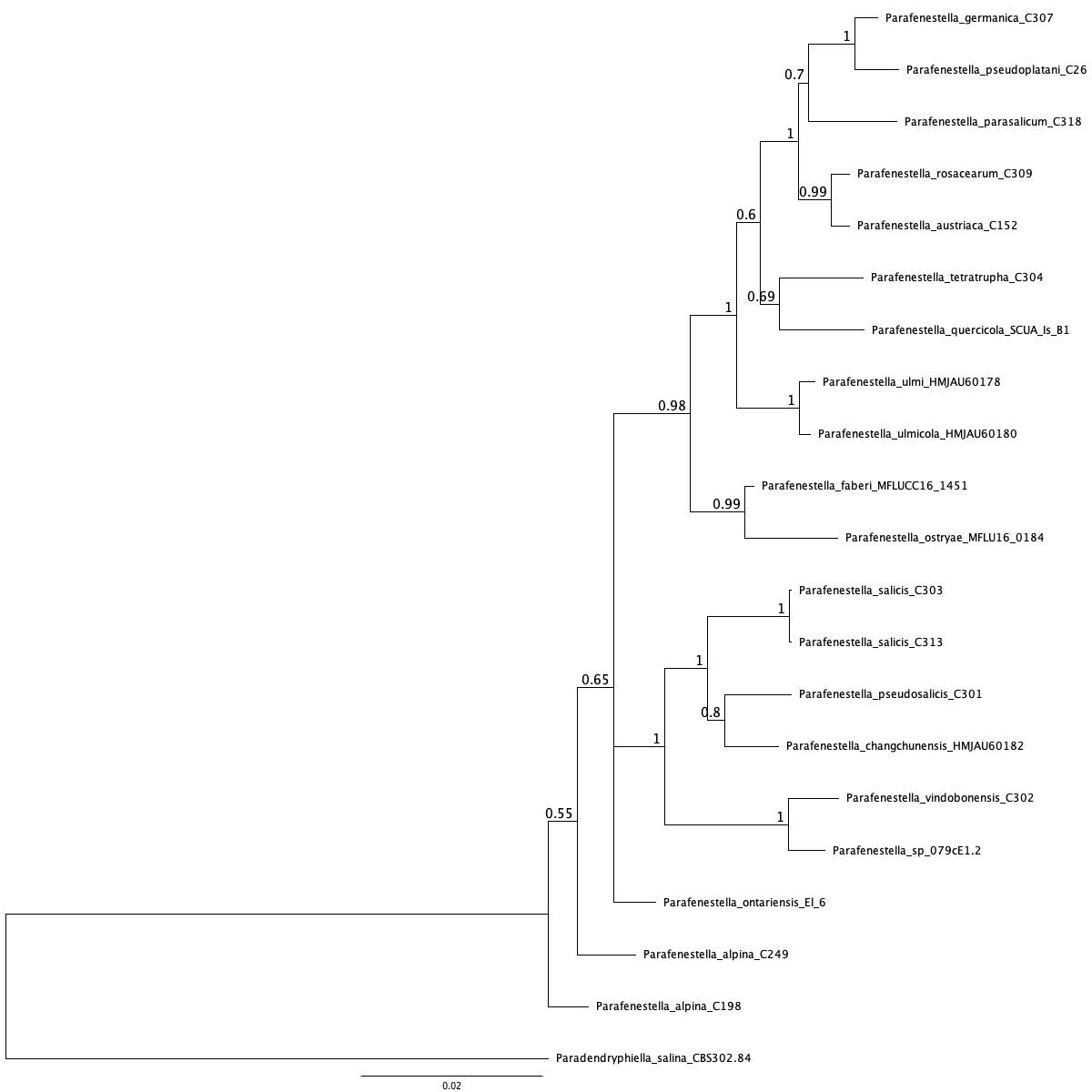


Figure S 1. Phylogenetic tree from Bayesian analysis with MrBayes, showing the placement of the isolate 079cE1.2.


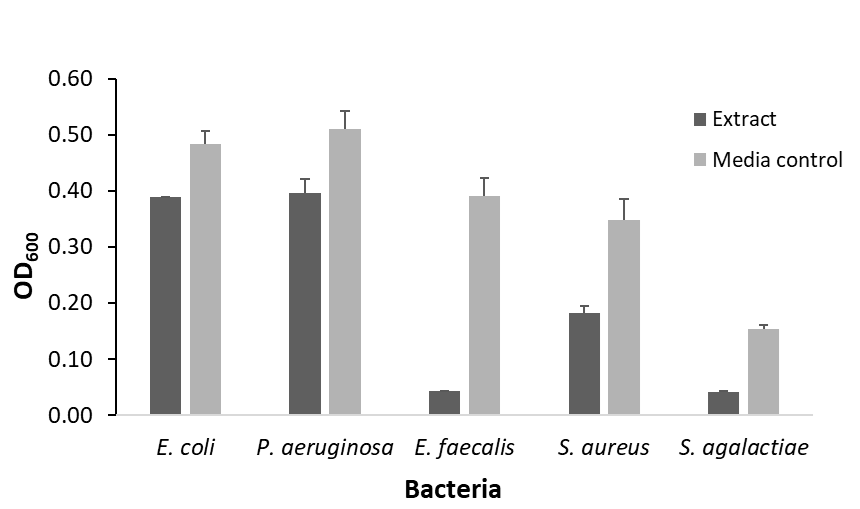


Figure S 2. Initial screening of antibacterial activity of the fungal extract towards five pathogenic bacteria.


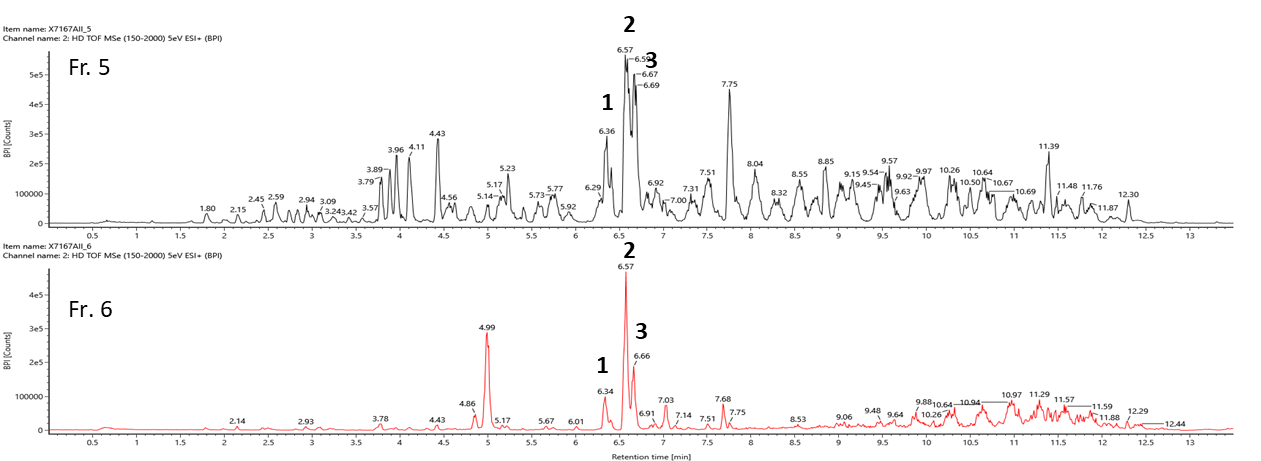


Figure S 3. UHPLC-HRMS base peak ion chromatogram of fractions 5 (Fr.5) and 6 (Fr.6) showing the presence of compounds 1, 2, and 3.

# Isolation of compounds from the fungus 079cE1.2

## First purification step

Fractions Fr.5 and Fr.6 were selected for compound isolation based on their distinctive chemical profiles and the presence of potentially bioactive compounds. Due to their similarity in UHPLC-HR-MS^E^ profiling, the fractions were combined before the isolation and subjected to preparative high-performance liquid chromatography-mass spectrometry (prep HPLC-MS) (Waters Auto Purification HPLC system consisting of a 600 HPLC pump with a 2767 sample manager, a 2996 PDA detector, and a 3100-mass spectrometer operated by MassLynx version 4.1). The column used to purify the compounds was a Sunfire^®^ C18 OBD™ prep column (250 × 10 mm, 5 μm) with the mobile phases A (0.1% formic acid in water) and B (0.1% formic acid in acetonitrile) in the first purification step. The following gradient was applied at a flow rate of 6 mL min^−1^: 0 to 19 minutes (53% B), 20 to 23 minutes (100% B), and 23.1 to 25 minutes (53% B) to isolate three targeted compounds (**1**, **2**, and **3**).

## Monitoring the stability of the compounds

The stability of **1**–**3** was monitored over time under controlled conditions, with storage at 5 °C to assess any changes in their chemical composition or formation of degradation products. The compounds were analyzed using UHPLC-HR-MS^E^, which consisted of a UPLC^®^ BEH C18 column (100 mm × 2.1 mm, 1.7 µm) with solvent A (0.1% formic acid in water) and solvent B (0.1% formic acid in acetonitrile) as mobile phases, at a flow rate of 0.45 mL min^−1^.

## Second purification step

Compounds **1** and **2** were further purified (second purification step) using XSelect^TM^ CSH^TM^ Phenyl-hexyl Prep column (250 mm × 10 mm, 100 Å, 5 µm) with isocratic elution at 40% B for 24 minutes followed by a 100% B washing step with a flow rate of 6 mL min^−1^ to yield 1.2 mg of **2**. Compound **3** was also purified with the same phenyl-hexyl prep column but with isocratic elution at 38% B for 27 minutes, followed by a 100% B washing step.


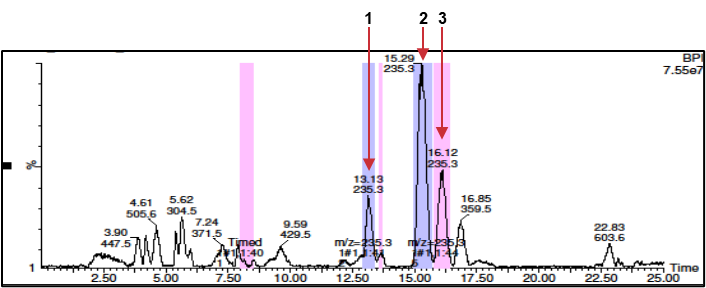


Figure S 4. Preparative HPLC-MS base peak ion chromatogram of Fr.5. First round purification of 1, 2, and 3.


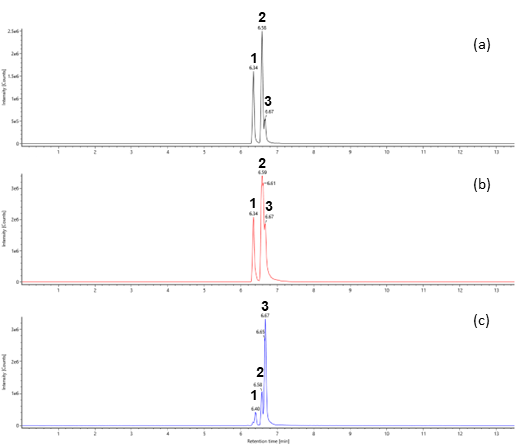


Figure S 5. UHPLC-HR-MS base peak ion chromatograms of isolated compounds 1, 2, and 3 to assess the stability of the compounds. (a) Chromatogram of 1 shows the detection of all three compounds. (b) Chromatogram of 2 shows the presence of all three compounds. (c) Chromatogram of 3 shows the presence of all three compounds. It suggests that these compounds are unstable and interconvertible with each other when exposed to elevated temperature (40 °C).


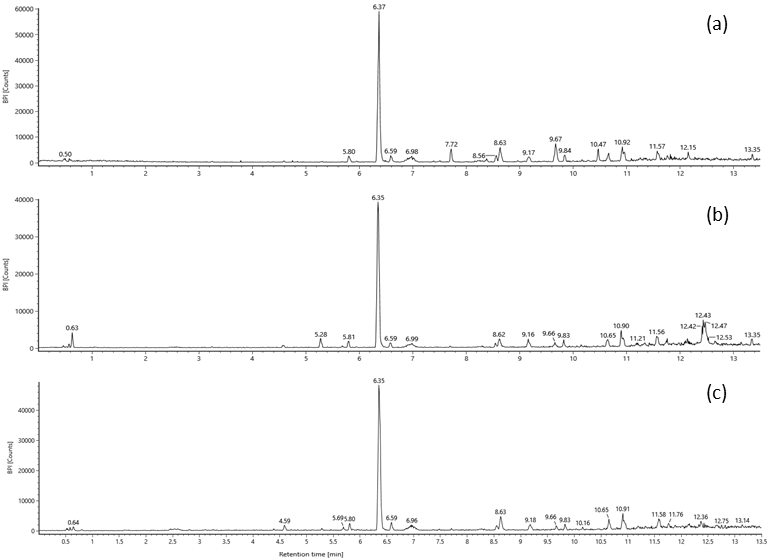


Figure S 6. UHPLC-HR-MS base peak ion chromatogram showing the stability of 1 on Day 1 (a), Day 2 (b), and Day 5 (c) when stored at a low temperature (5 °C).


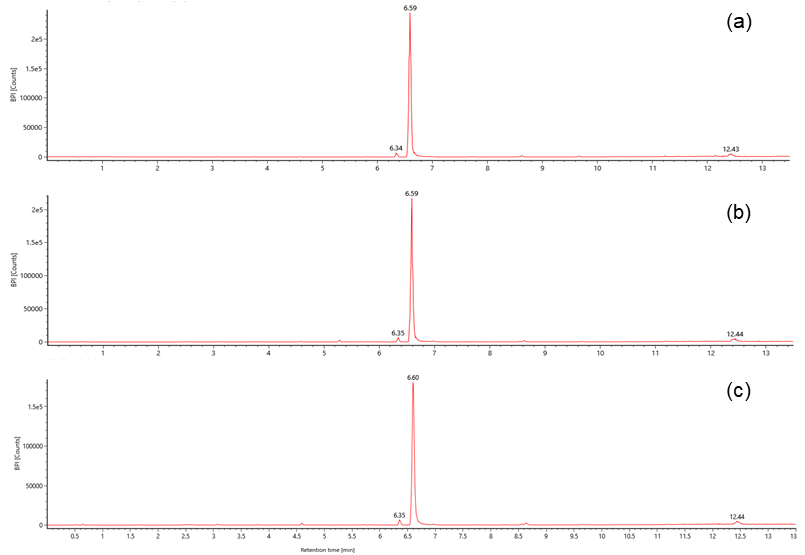


Figure S 7. UHPLC-HR-MS base peak ion chromatogram showing the stability of 2 on Day 1 (a), Day 2 (b), and Day 5 (c) when stored at a low temperature (5 °C).


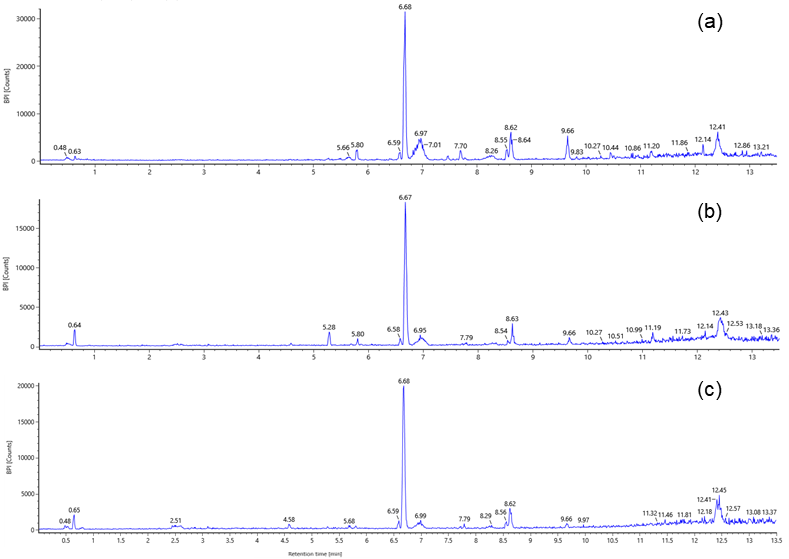


Figure S 8. UHPLC-HRMS base peak ion chromatogram showing the stability of 3 on Day 1 (a), Day 2 (b), and Day 5 (c) when stored at a low temperature (5 °C).


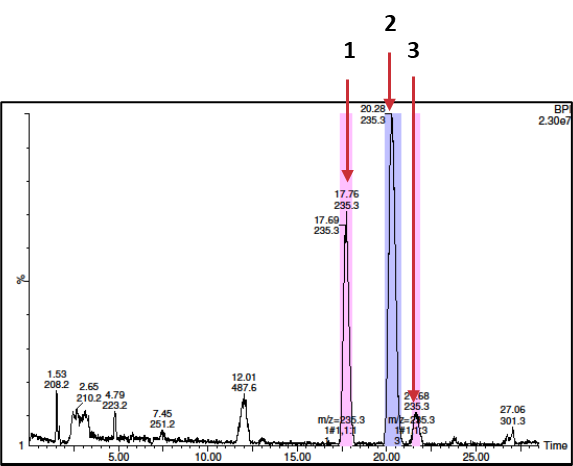


Figure S 9. Preparative HPLC-MS base peak ion chromatogram of 1 (Second round purification).


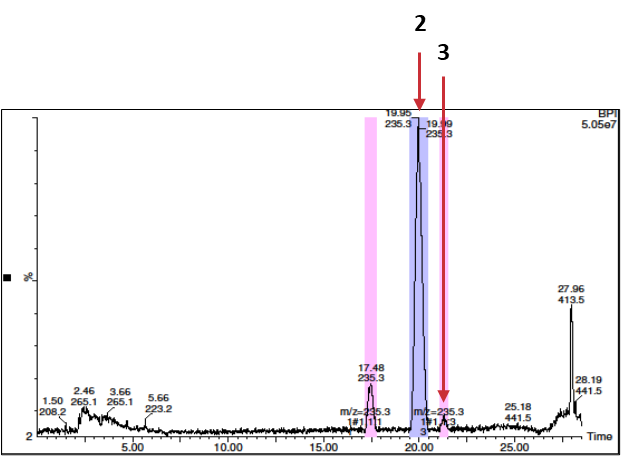


Figure S 10. Preparative HPLC-MS base peak ion chromatogram of 2 (Second round purification).


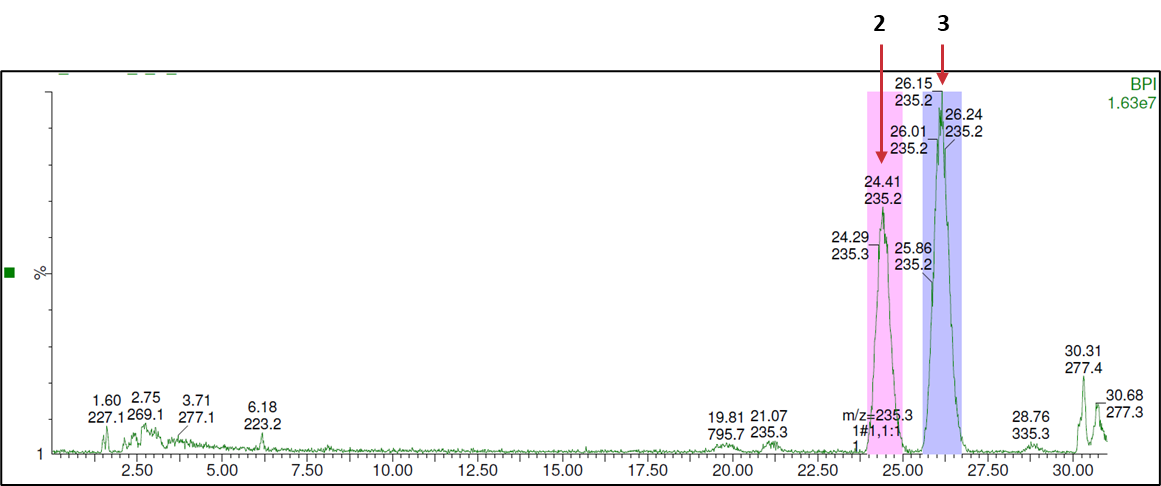


Figure S 11. Preparative HPLC-MS base peak ion chromatogram of 3 (Second round purification).


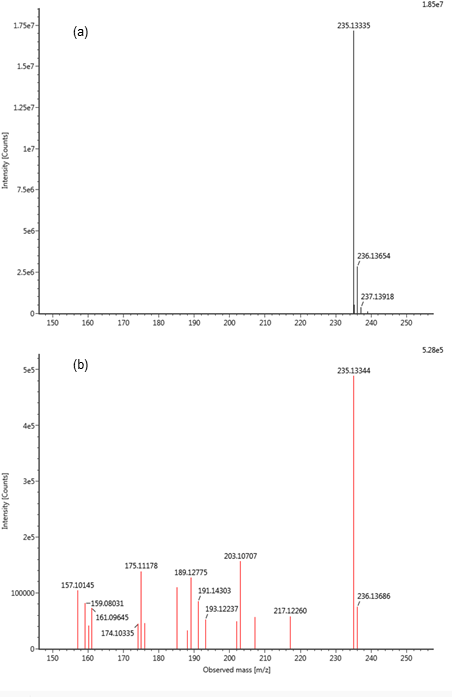


Figure S 12. HRESIMS of 1 at (a) low collision energy (b) high collision energy in ESI^+^ mode.

# Structure elucidation of isolated compounds

The structures of **2** and **3** were determined using 1D and 2D NMR experiments. NMR spectra were acquired in DMSO-*d*_6_ at 25 °C on a Bruker AVANCE III spectrometer operating at 600 MHz for ^1^H, which was equipped with a cryogenically enhanced TCI probe. The ^1^H and ^13^C nuclei were observed at 600 MHz and 150 MHz, respectively. Chemical shifts (δ) were expressed in parts per million (ppm) relative to the residual solvent signal, with coupling constants *J* in Hz.

Compound **2** was obtained as a light reddish-brown solid. The molecular formula of **2** was calculated to be C_14_H_18_O_3_, based on HRESIMS *m/z* 235.1335 [M+H]^+^ (calcd. for 235.1334), corresponding to six degrees of unsaturation that suggested a presence of a polyene or aromatic structure. The complete structure of **2** was elucidated by the interpretation of 1D (^1^H and ^13^C) and 2D (HSQC, HMBC, COSY, and ROESY) NMR spectra. The ^1^H NMR spectrum (Table S3) of **2** showed the presence of four methyl groups (δ_H_, 1.74, 1.80, 1.84, and 2.05), one methoxy (δ_H_ 3.95), three methine protons (one vinylic (δ_H_ 5.68 (q), *J* = 7.0 Hz) and two aromatic protons (δ_H_ 6.54, and 6.81)). The ^13^C NMR spectrum (Table S3) showed 14 carbon signals, attributable to four methyl carbons (δ_C_, 8.67, 13.8, 14.0, 16.4), one methoxy (δ_C_ 56.8), nine sp^2^ hybridized carbons (two tetrasubstituted olefinic (δ_C_ 93.6, 100.0), four olefinic (δ_C_ 124.6, 129.4, 132.6, 135.2), and three carbonyl-like (δ_C_ 165.6, 166.2, 159.7)). The HSQC spectrum supported the interpretation of all these carbon signals. The observed HMBC correlation signals are shown in Figure S13. The correlations observed from methyl protons H_3_-14 (δ_H_ 6.50) to C-3 (δ_C_ 100.0), C-2 (δ_C_ 165.6), and C-4 (δ_C_ 166.2); from methoxy proton H_3_-4 (δ_H_ 3.95) to C-4; and from H-5 (δ_H_ 6.54) to C-2, C-4, and C-6 (δ_C_ 159.7) allowed the assignment of an α-pyrone moiety. This confirmed the attachment of the methyl (H_3_-14) and methoxy groups to C-3 and C-4, respectively. Moreover, HMBC correlations from H-5 to C-7 (δ_C_ 124.6) and from methyl proton (CH_3_-13, δ_H_ 13.8) to C-6 and C-7 confirmed the attachment of a diene substituent at C-6. The key HMBC correlations shown in Figure S13 indicate the partial structure of the diene substituent. Furthermore, the partial structure of the diene substituent and configurations of the double bond at C-7 and C-9 were deduced by the ROESY experiment. The ROE correlations between H-5/H_3_-13, H-8/H-10, and H_3_-12/H_3_-16 and the absence of other correlations H_3_-13/H-8 and H_3_-12/H-10 provided evidence for the *E*-configuration at ∆^7^ and ∆^9^-double bonds. Hence, the structure of **2** was confirmed to be phomenin A (polypropionate α-pyrones). Phomenin A has been previously reported and characterized using NMR data obtained from different NMR solvents (Ivanova et al. 2010, Pedras & Chumala 2005, Pedras et al. 1994, Tringali et al. 1993).

Compound **3** was isolated as a light reddish-brown solid. It is an isomer of **2**, as indicated by the same protonated mass (*m/z* 235.1335 [M+H]^+^) and the molecular formula (C_14_H_18_O_3_) determined by HRESIMS analysis. ^1^H and ^13^C NMR spectroscopic data of **2** were similar to **3** except for a slightly deshielded methyl carbon (CH_3_-12, δ_C_ 23.2) in the ^13^C spectrum of **3**, as shown in Table S3. A ROESY experiment provided evidence that they differ only in the stereochemistry of the C-9 double bond. The configuration of the ∆^9^-double bond was assigned as 9*Z* based on ROE correlations between H_3_-13/H_3_-12, H_3_-12/H-10, H-8/H-11, and the absence of the H-8/H-11 correlation. Consequently, the structure of **3** was confirmed to be phomenin B (Tringali et al. 1993).

Figure S 13. Key HMBC (blue arrow) and ROESY (red arrow) correlations of 2 and 3.

## Phomenin A (2)

Colourless residue; C_14_H_18_O_3_; HRESIMS *m/z* 235.1335 [M + H]^+^ (calcd. for C_14_H_19_O_3_^+^, 235.1334); Observed CCS value 159.88 Å^2^. ^1^H NMR (DMSO-*d_6_*, 600 MHz): δ 6.81 (s, 1H), 6.54 (s, 1H), 5.68 (q, *J* = 7.0 Hz, 1H), 3.95 (s, 3H), 2.05 (s, 3H), 1.84 (s, 3H), 1.80 (s, 3H), 1.74 (d, *J* = 7.0 Hz, 3H). ^13^C NMR (DMSO-*d_6_*, 150 MHz): δ 166.2, 165.6, 159.7, 135.2, 132.7, 129.4, 124.6, 100.0, 93.6, 56.7, 16.4, 14.0, 13.8, 8.6.

Phomenin B (**3**)

Colourless residue; C_14_H_18_O_3_; HRESIMS *m/z* 235.1332 [M + H]^+^ (calcd. for C_14_H_19_O_3_^+^, 235.1334); Observed CCS value 159.46 Å^2^. ^1^H NMR (DMSO-*d_6_*, 600 MHz): δ 6.78 (s, 1H), 6.58 (s, 1H), 5.51 (q, *J* = 7.0 Hz, 1H), 3.96 (s, 3H), 1.88 (s, 3H), 1.82 (s, 3H), 1.81 (s, 3H), 1.52 (d, *J* = 6.9 Hz, 3H). ^13^C NMR (DMSO-*d_6_*, 150 MHz): δ 166.0, 163.4, 158.7, 132.3, 130.7, 127.2, 124.4, 100.5, 94.0, 56.8, 23.2, 15.0, 13.9, 8.7.

Table S 3. ^1^H (600 MHz) and ^13^C (151 MHz) NMR data of 2 and 3 in DMSO-*d_6_*.

|  | Compound **2** | | Compound **3** | |
| --- | --- | --- | --- | --- |
| Position | δ_C_, type | δ_H_, mult. (*J* in Hz) | δ_C_, type | δ_H_, mult. (*J* in Hz) |
| 1 |  |  |  |  |
| 2 | 165.6, C |  | 163.4, C |  |
| 3 | 100.0, C |  | 100.5, C |  |
| 4 | 166.2, C |  | 166.02, C |  |
| 5 | 93.6, CH | 6.54, s | 93.97, CH | 6.58, s |
| 6 | 159.7, C |  | 158.7, C |  |
| 7 | 124.6, C |  | 127.2, C |  |
| 8 | 135.2, CH | 6.81, s | 130.7, CH | 6.78, s |
| 9 | 132.7, C |  | 132.3, C |  |
| 10 | 129.4, CH | 5.68, q (*J* = 7.0 Hz) | 124.4, CH | 5.51, q (*J* = 7.0 Hz) |
| 11 | 14.0, CH_3_ | 1.74, d (*J* = 7.0 Hz) | 14.98, CH_3_ | 1.52, d (*J* = 6.9 Hz) |
| 12 | 16.4, CH_3_ | 1.84, s | 23.2, CH_3_ | 1.82, s |
| 13 | 13.8, CH_3_ | 2.05, s | 13.9, CH_3_ | 1.88, s |
| 14 | 8.6, CH_3_ | 1.80, s | 8.7, CH_3_ | 1.81, s |
| 15 | 56.7, CH_3_ | 3.95, s | 56.8,CH_3_ | 3.96, s |


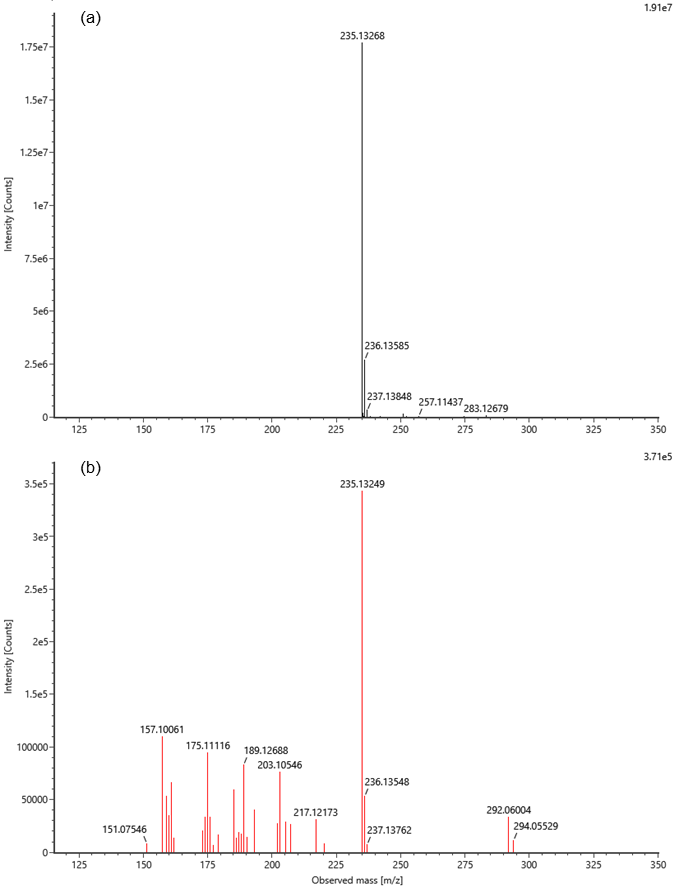


Figure S 14. HRESIMS of 2 at (a) low collision energy (b) high collision energy in ESI^+^ mode.


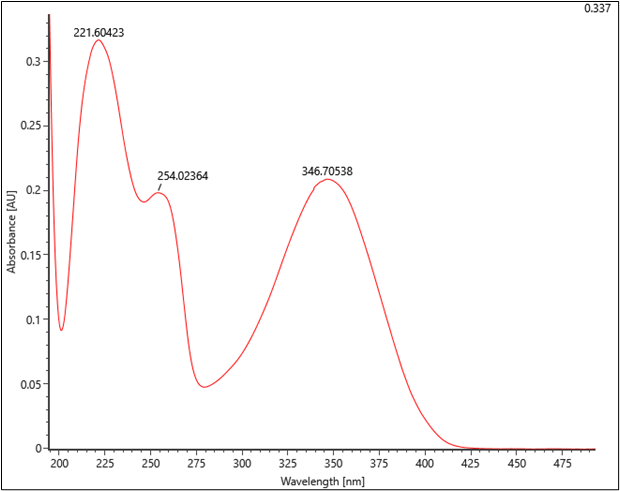


Figure S 15. UV-Vis spectrum of 2.


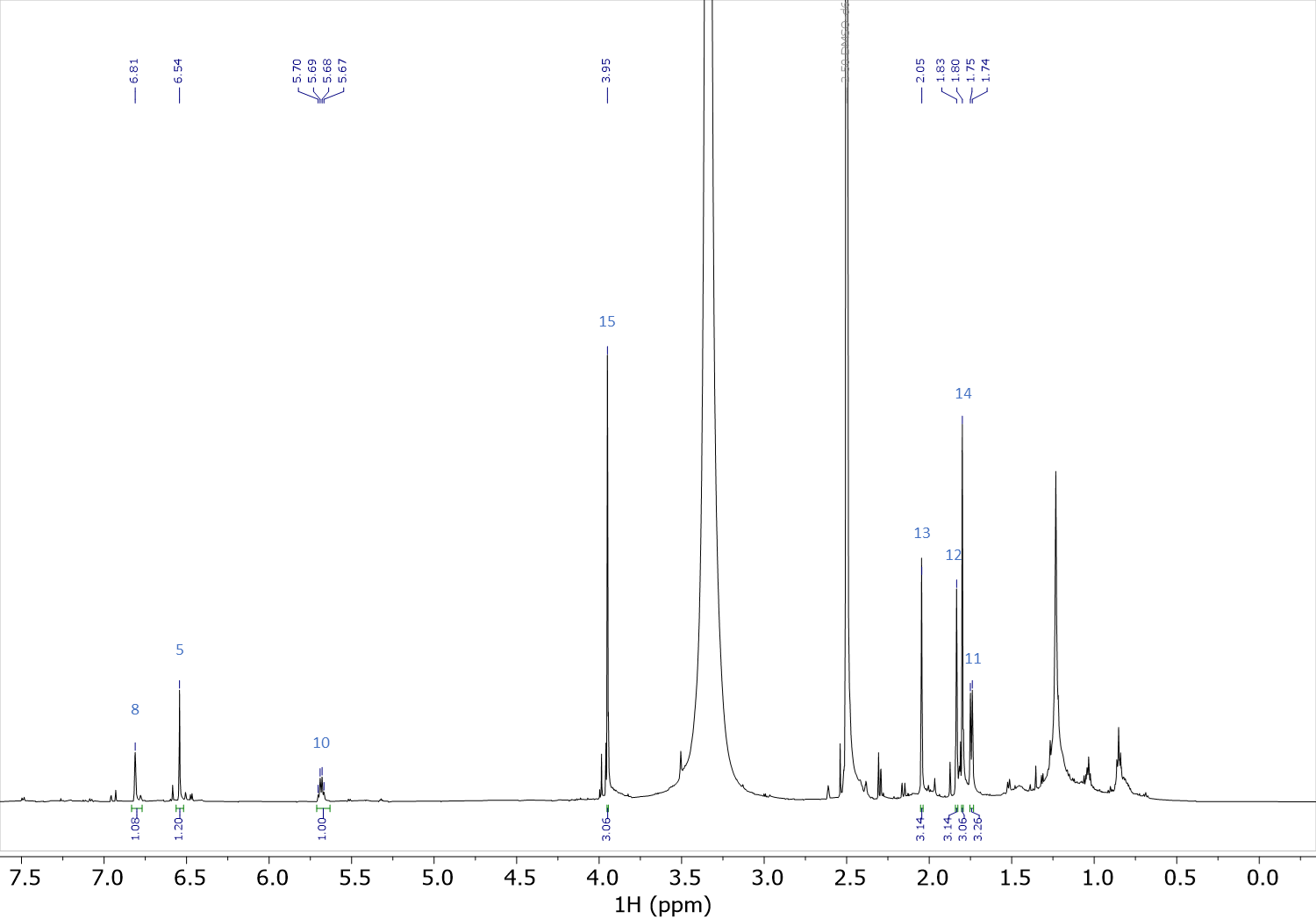


Figure S 16. ^1^H NMR spectrum (600 MHz, DMSO-*d_6_*) of 2.


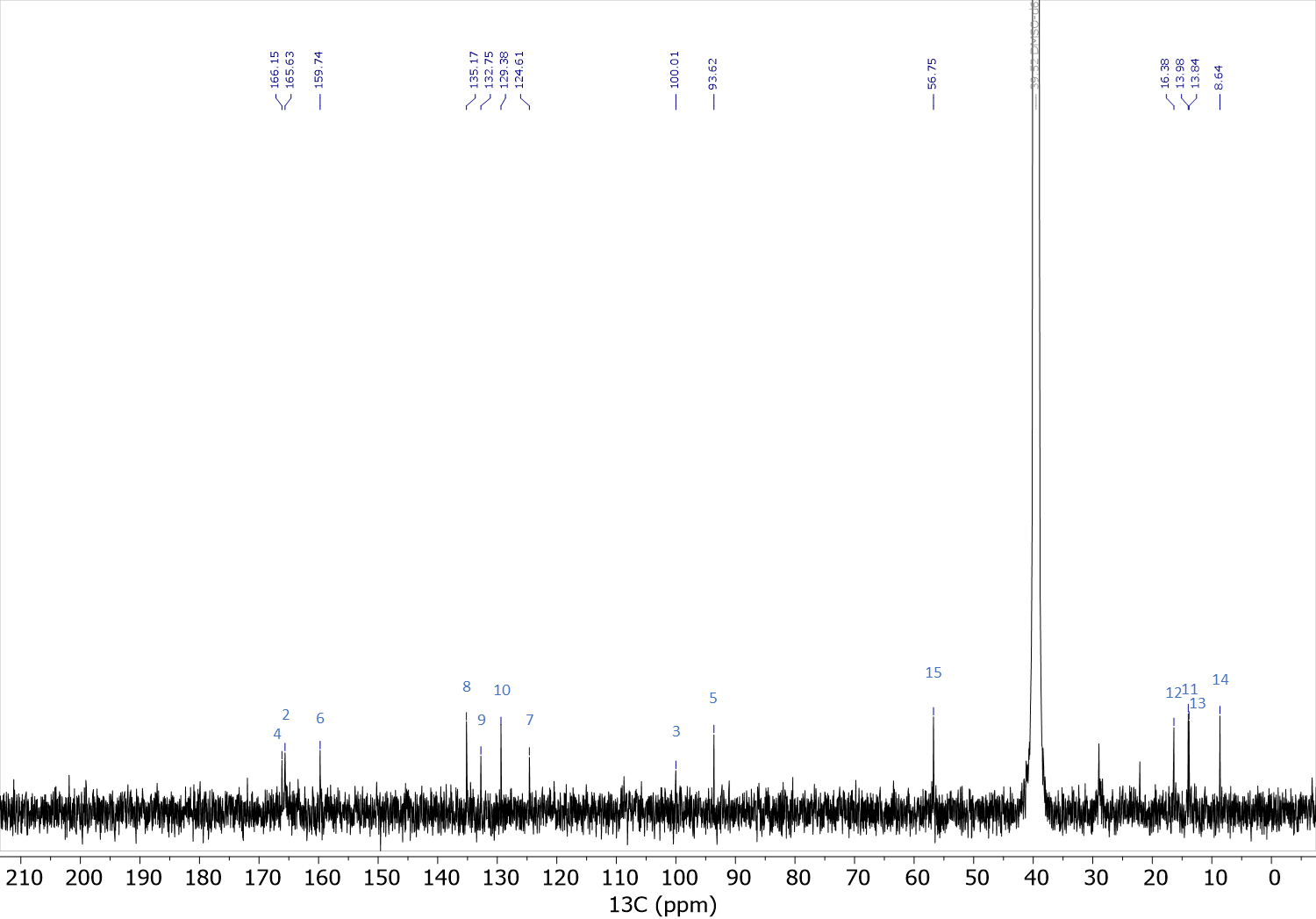


Figure S 17. ^13^C NMR spectrum (151 MHz, DMSO-*d_6_*) of 2.


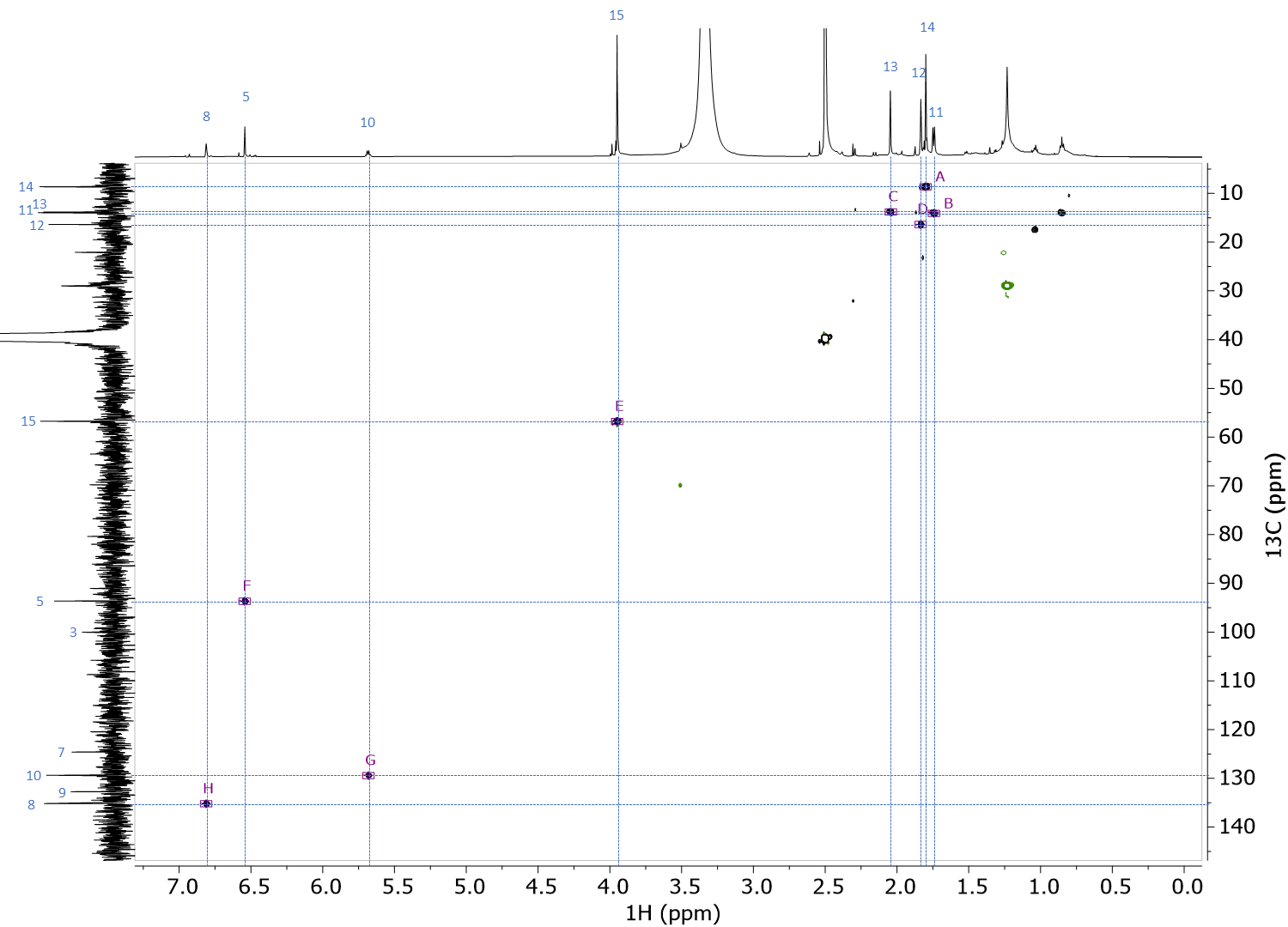


Figure S 18. HSQC spectrum of 2.


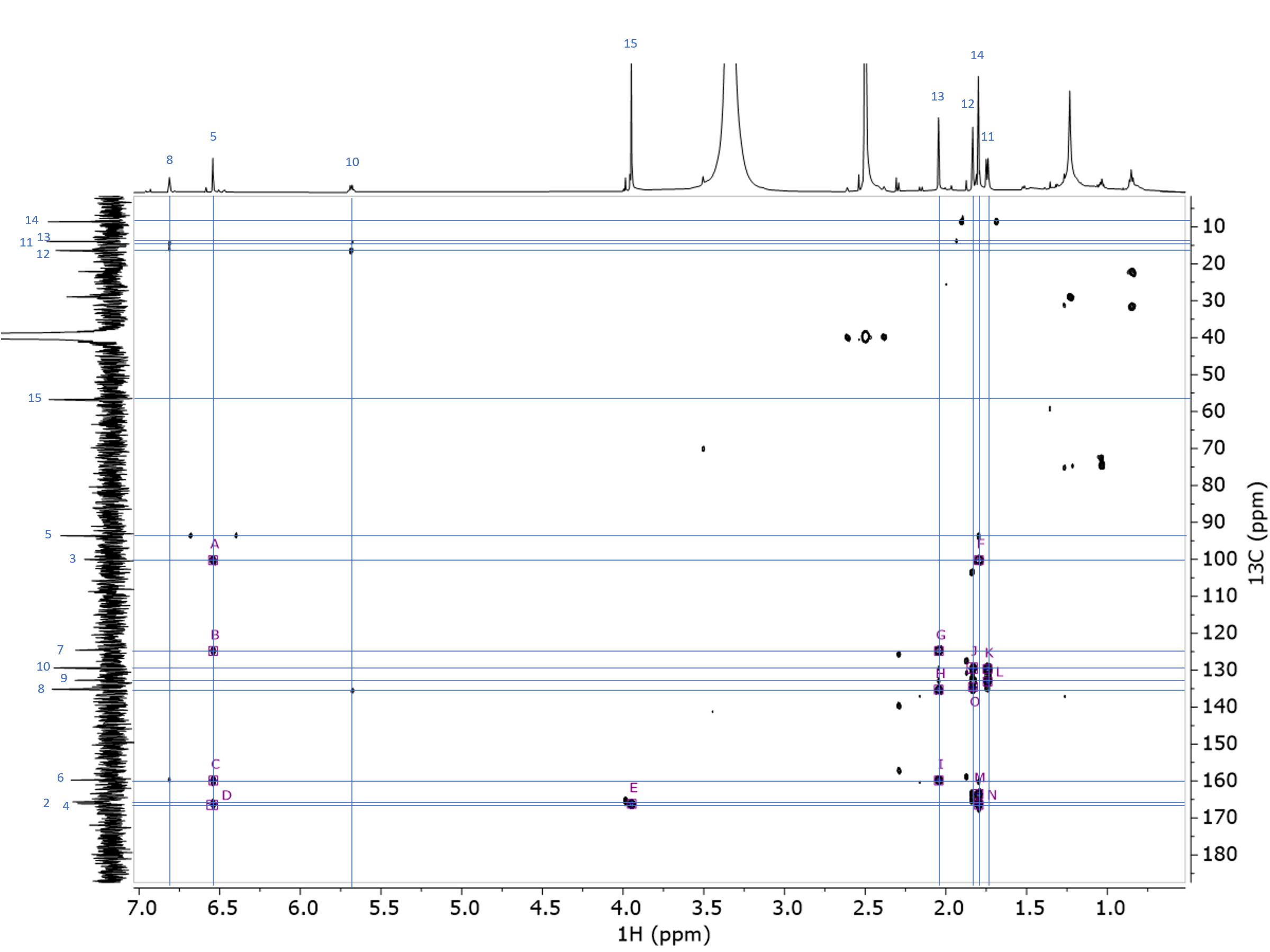


Figure S 19. HMBC spectrum of 2.


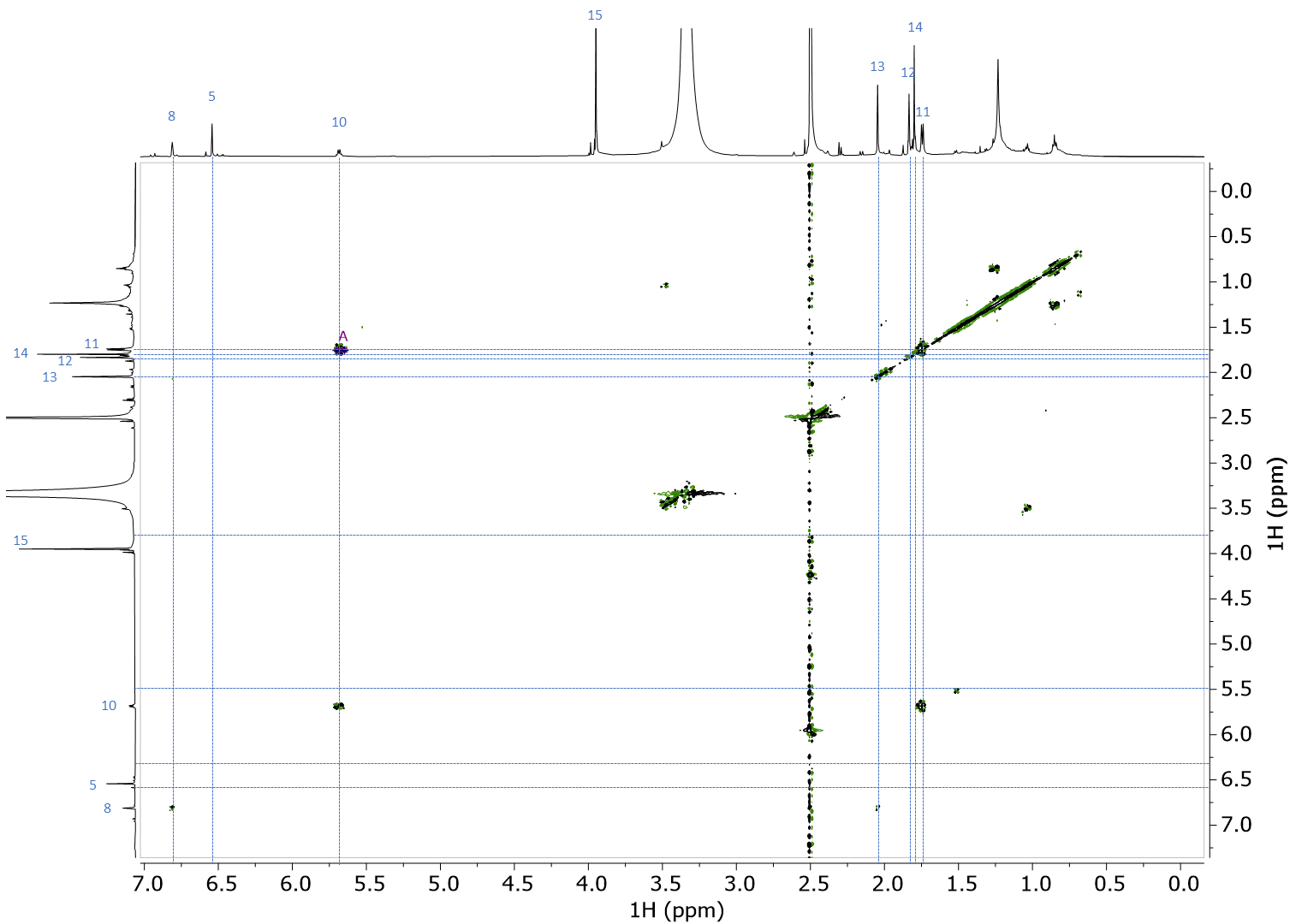


Figure S 20. ^1^H-^1^H COSY spectrum of 2.


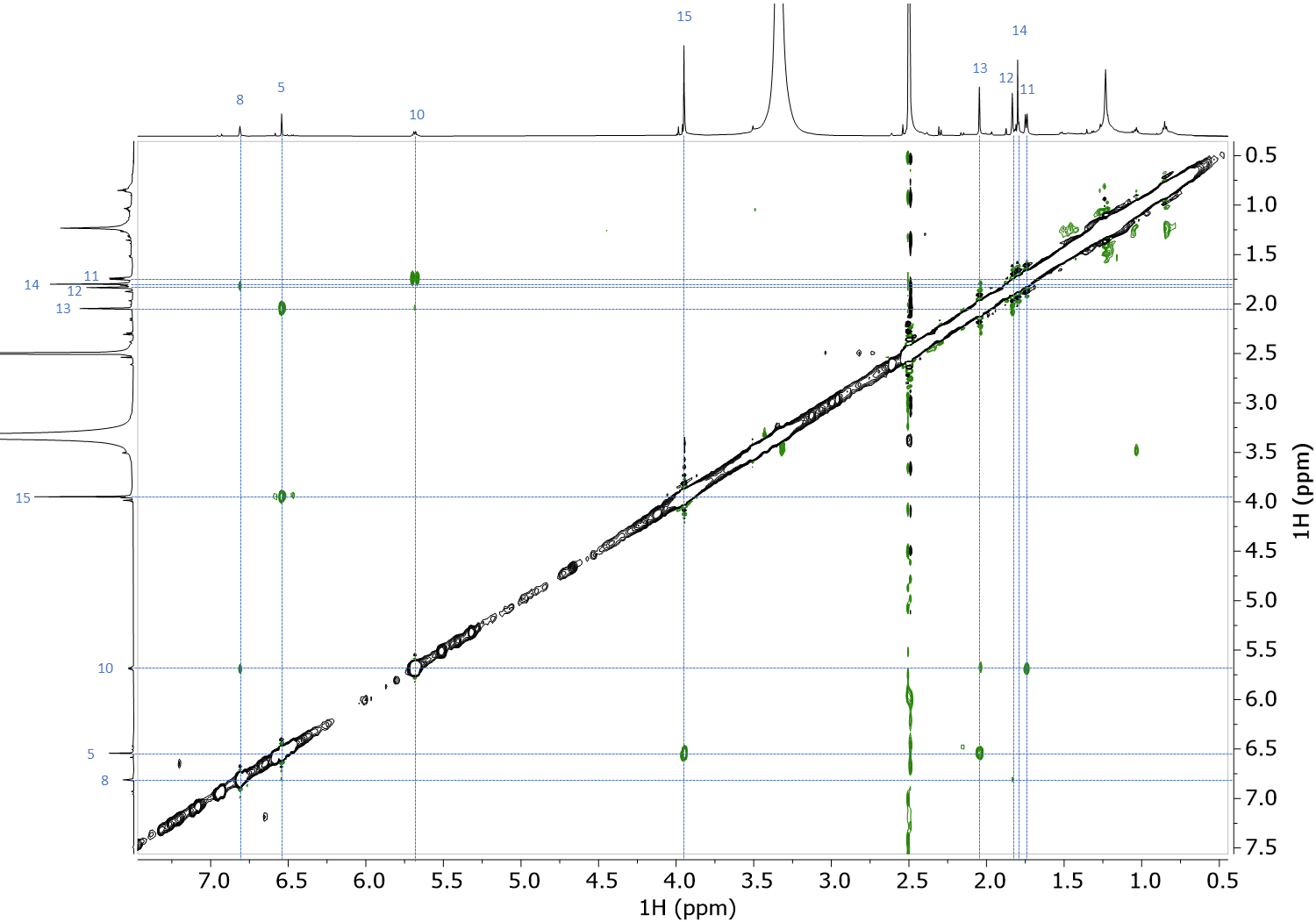


Figure S 21. ROESY spectrum of 2.


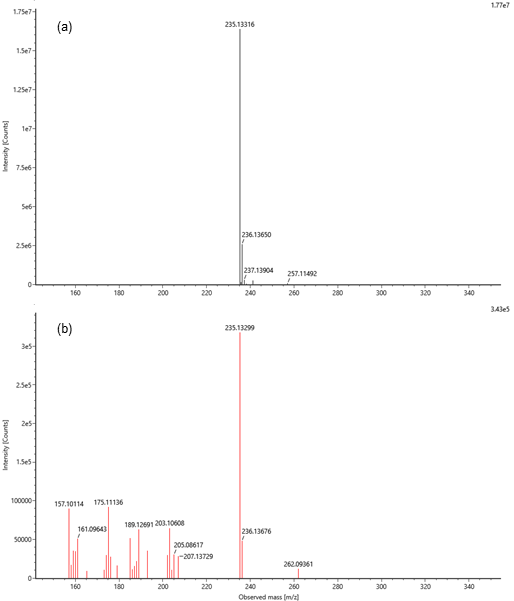


Figure S 22. HRESIMS of 3 at (a) low collision energy (b) high collision energy in ESI^+^ mode.


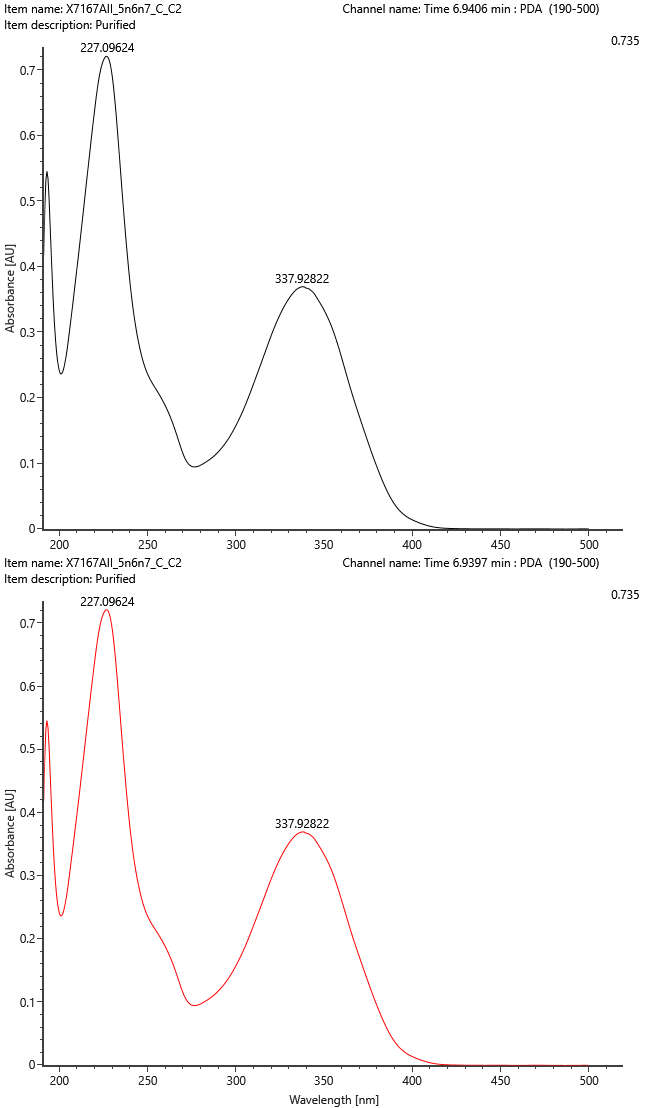


Figure S 23. UV-Vis spectrum of 3.


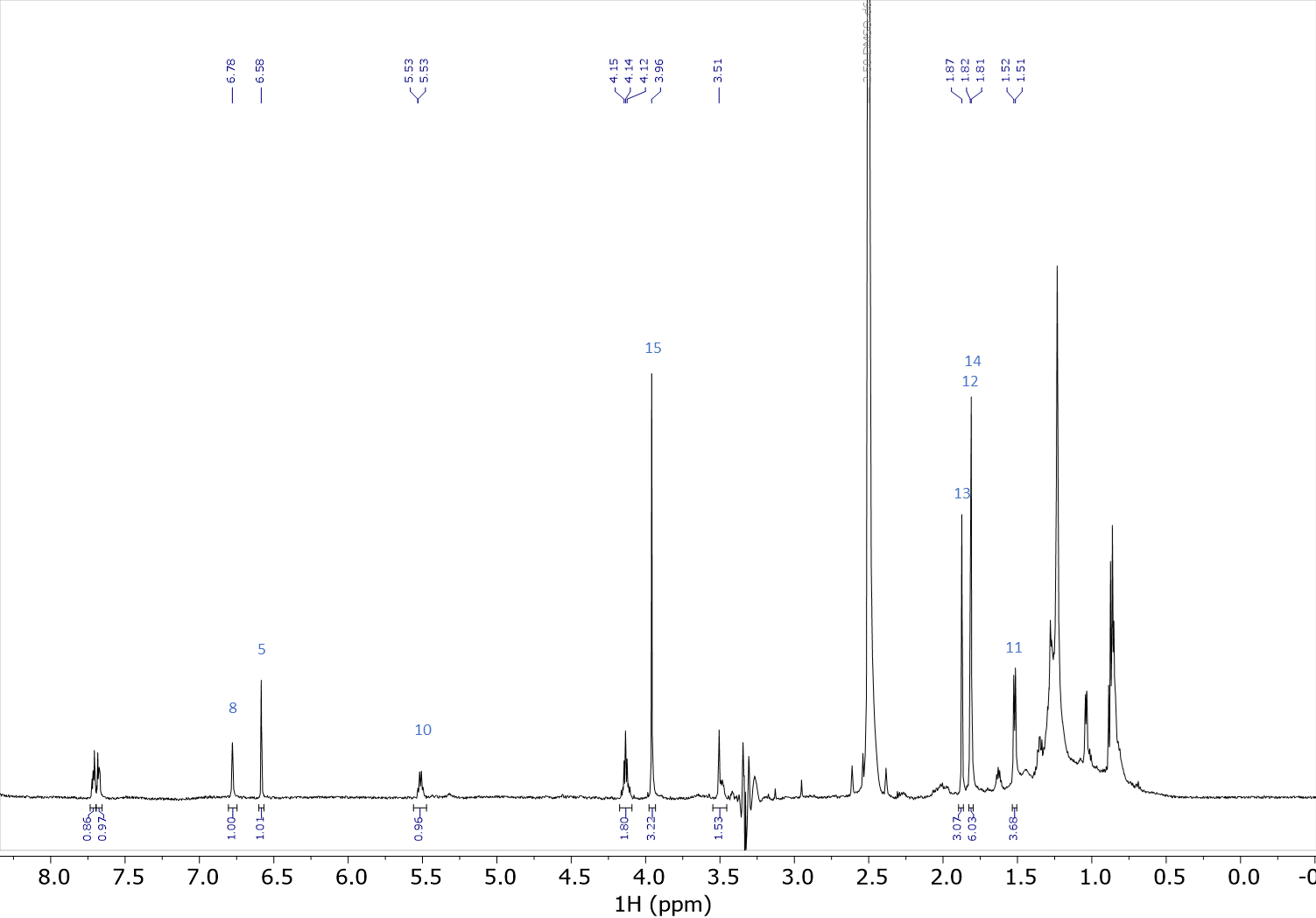


Figure S 24. ^1^H NMR of 3 (after the first round of purification).


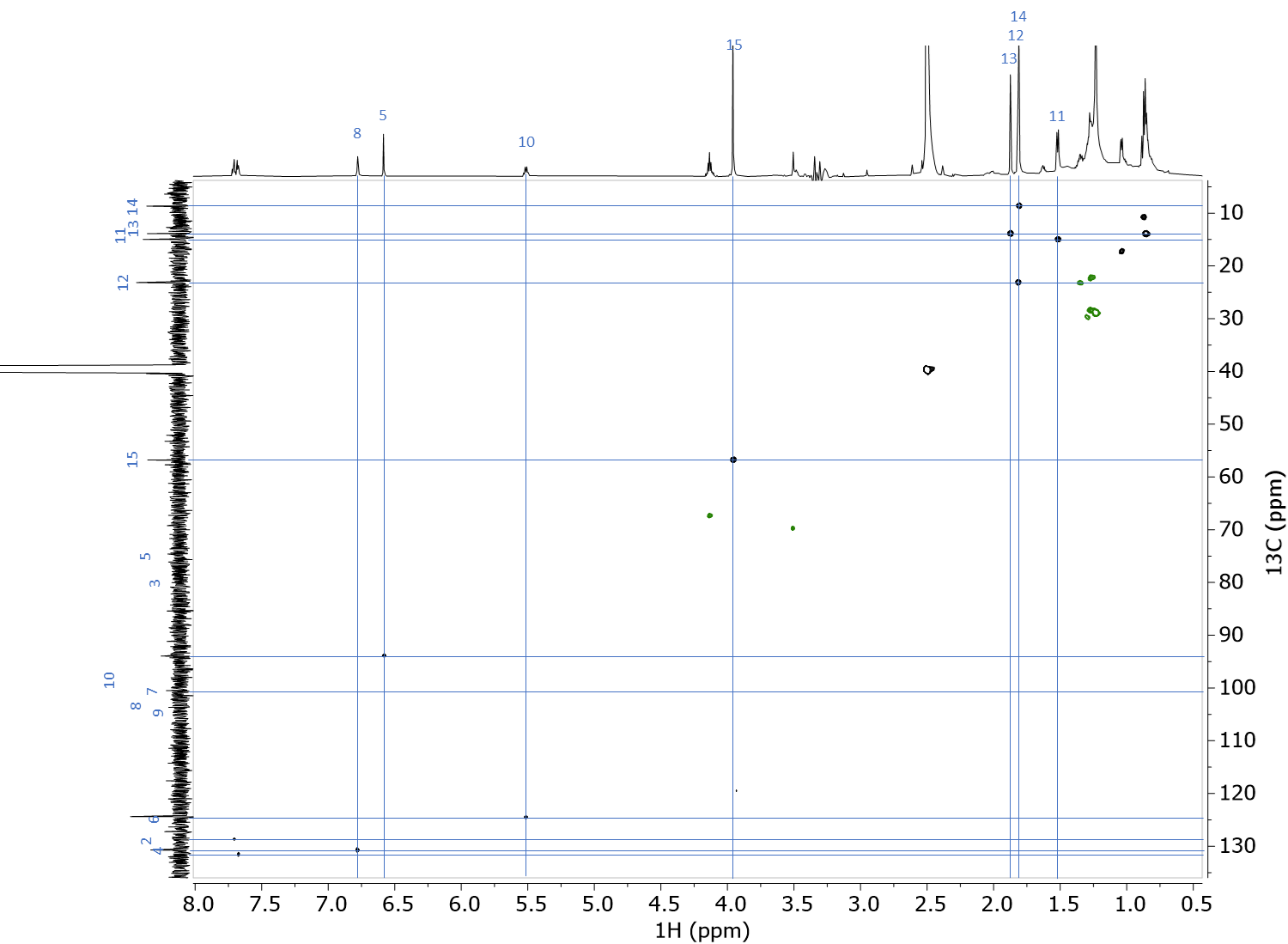


Figure S 25. HSQC of 3 (after the first round of purification).


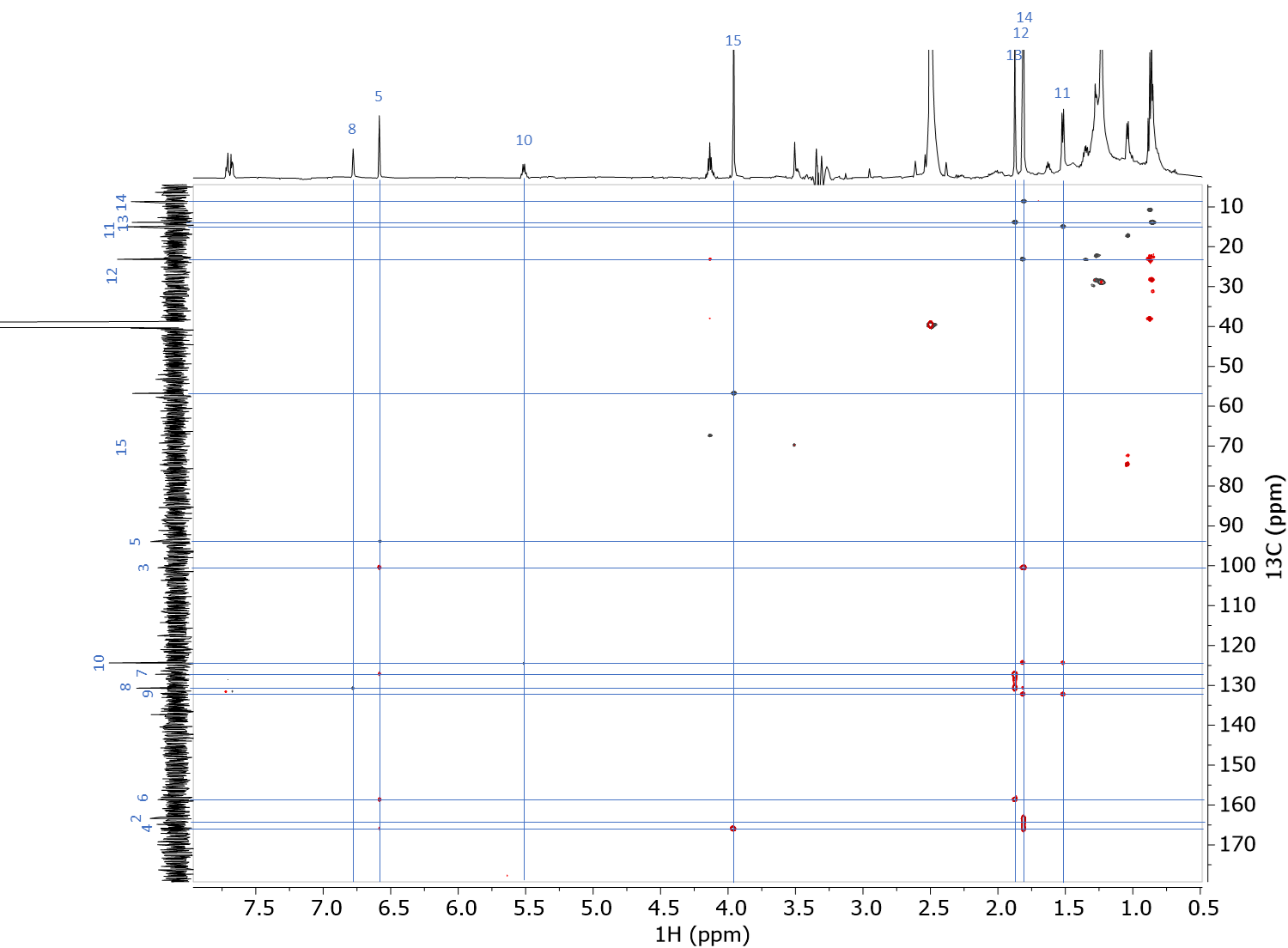


Figure S 26. HSQC + HMBC of 3 (after the first round of purification).


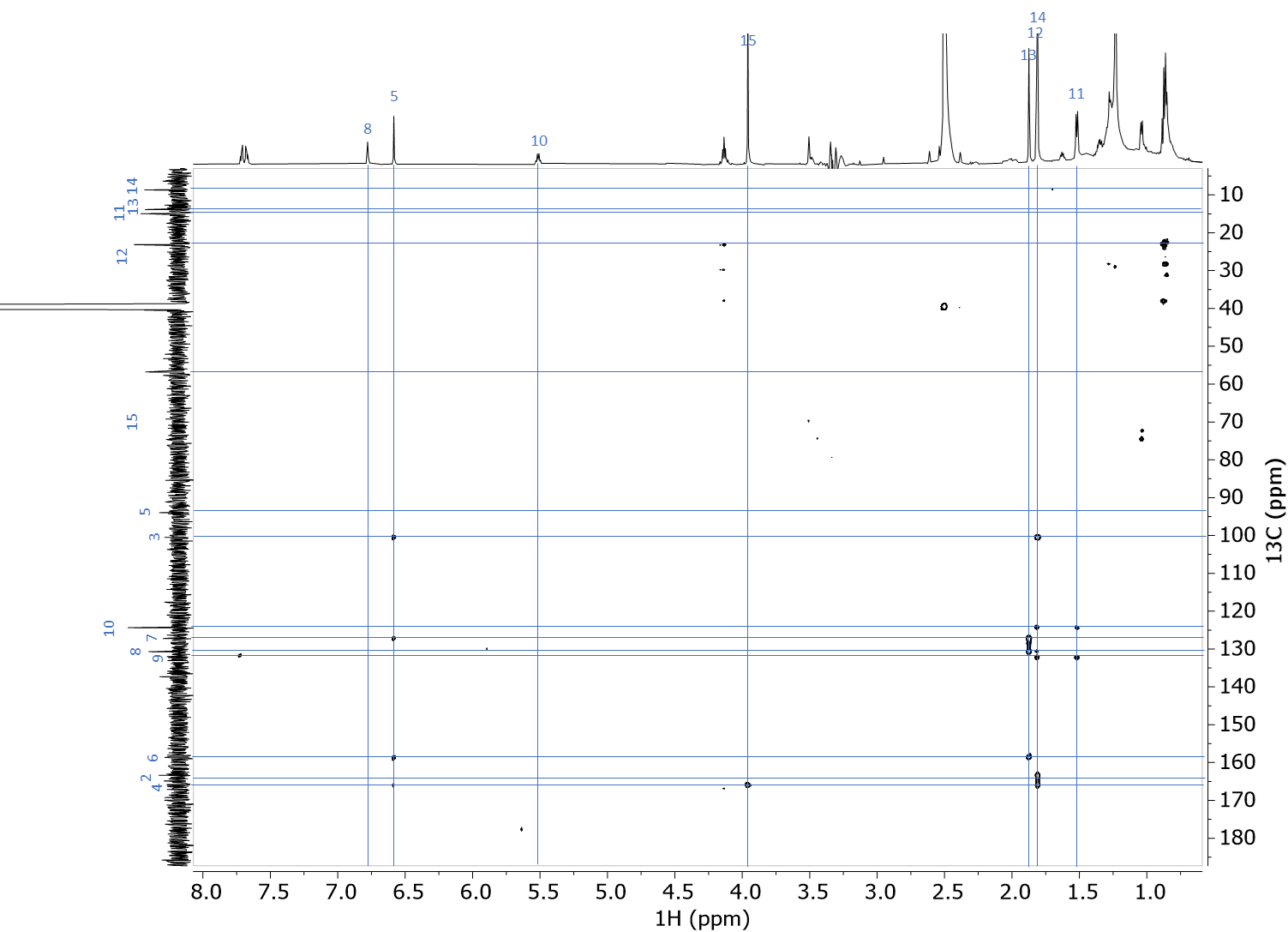


Figure S 27. HMBC of 3 (after the first round of purification).


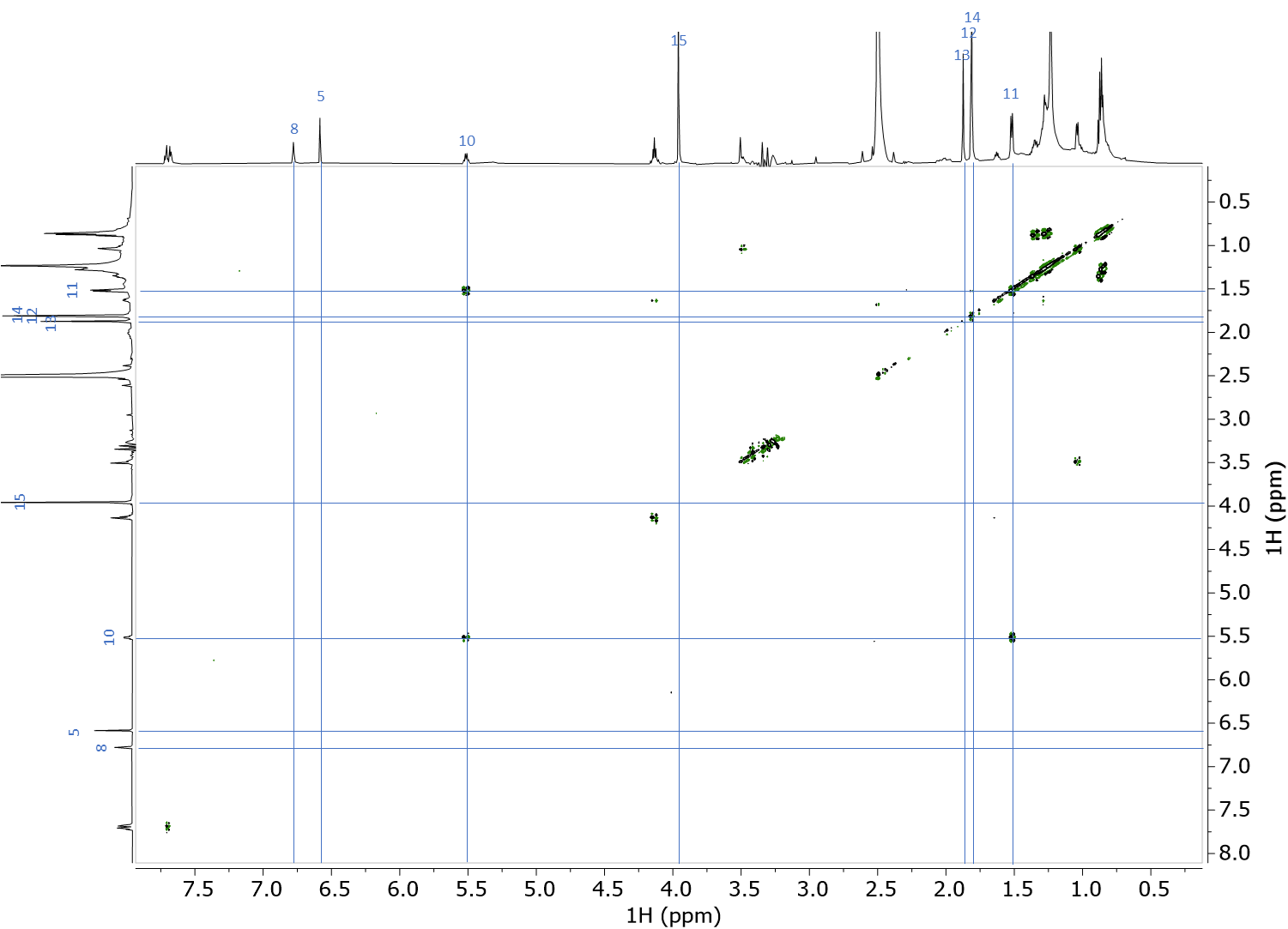


Figure S 28. COSY of 3 (after the first round of purification).


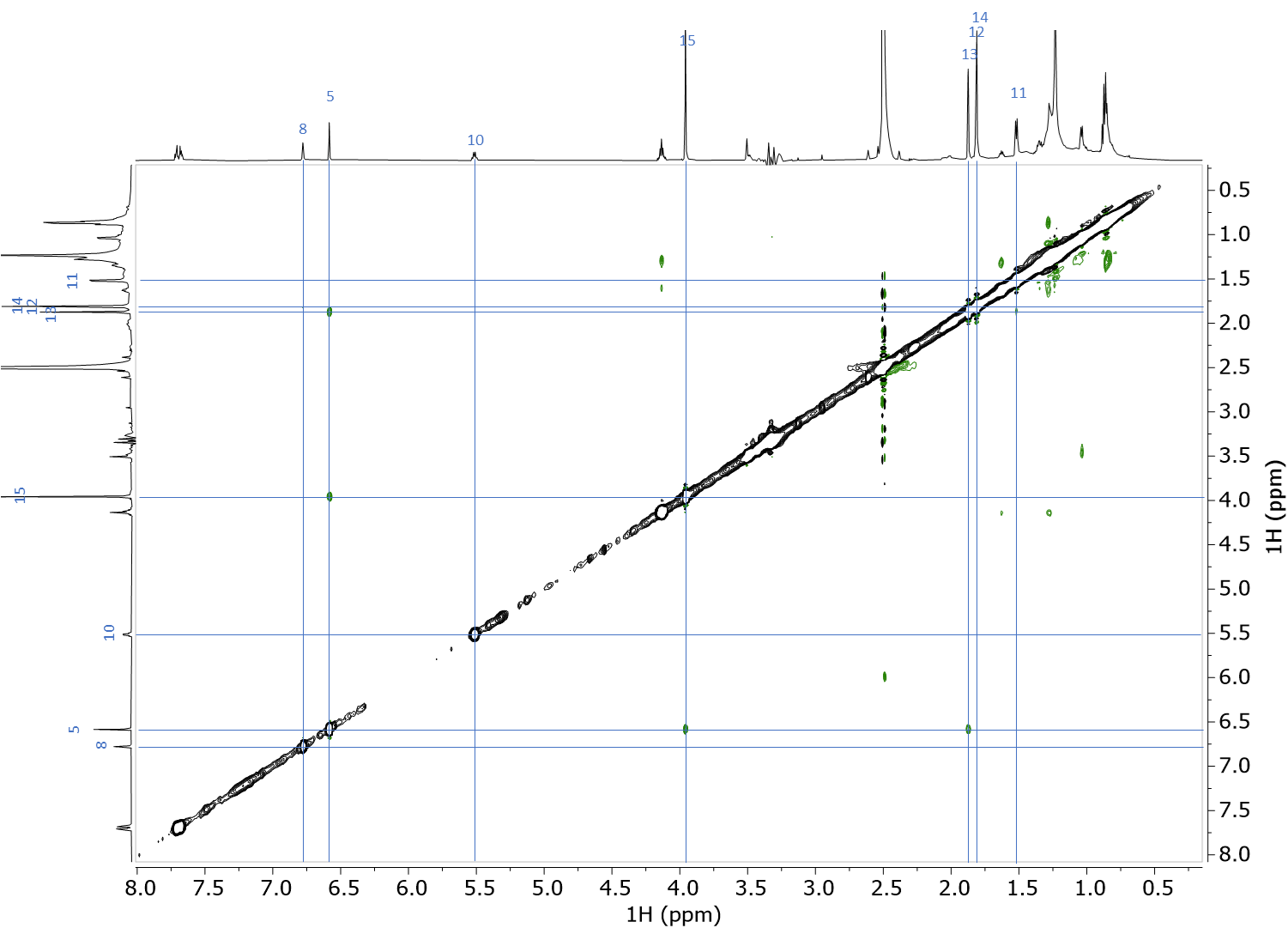


Figure S 29. ROESY of 3 (after the first round of purification).


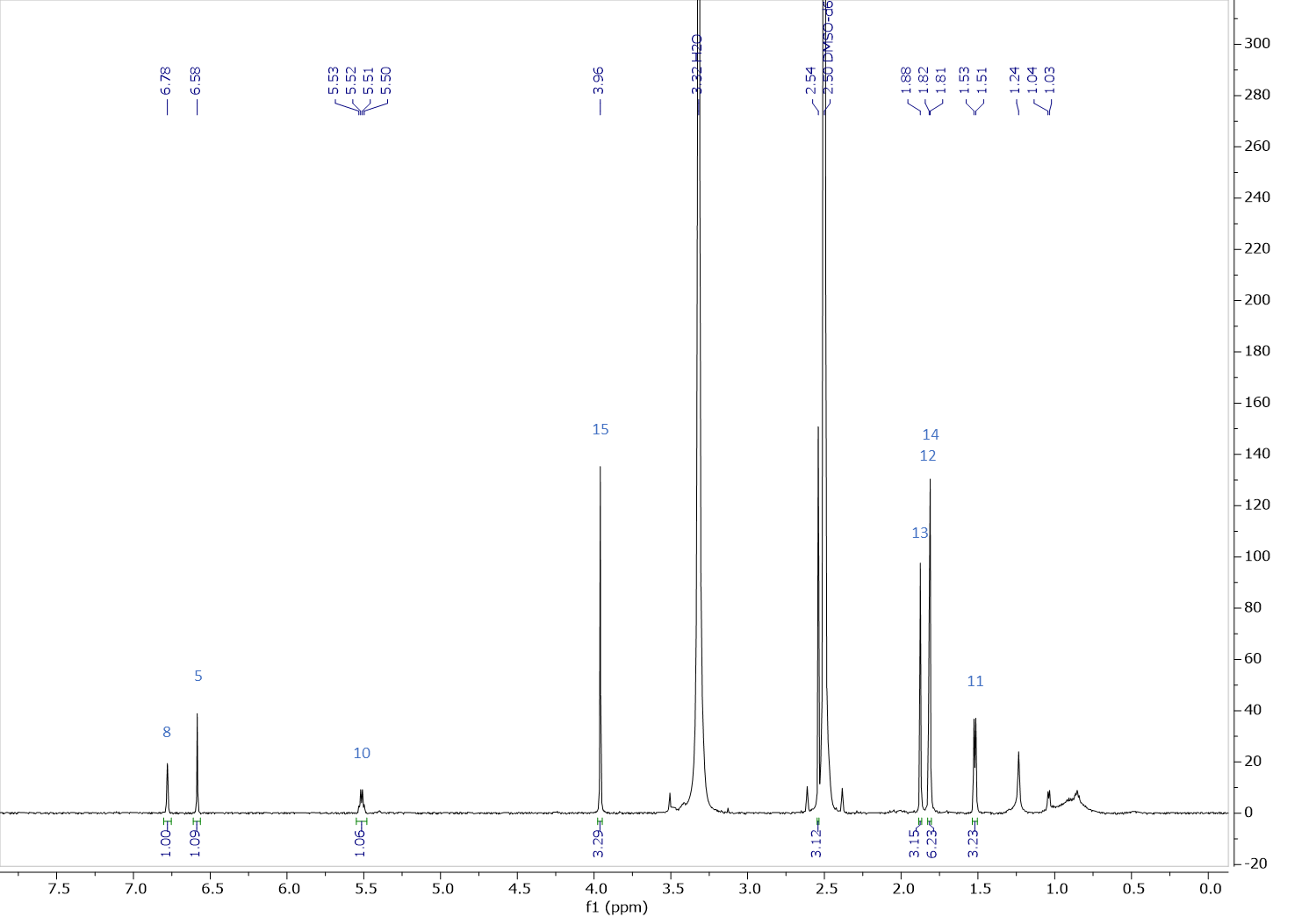


Figure S 30. ^1^H NMR of 3 (after the second round of purification).


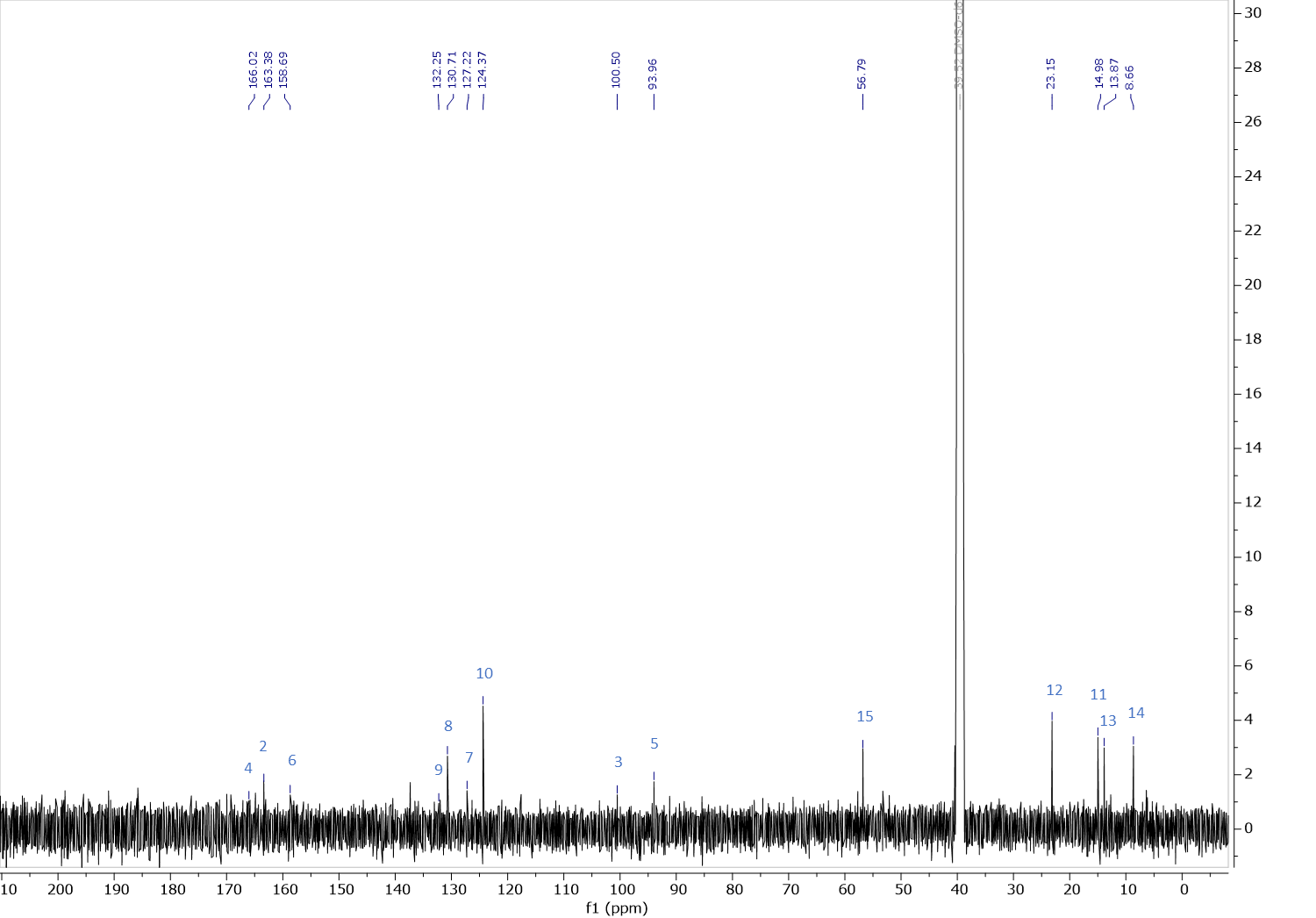


Figure S 31. ^13^C NMR of 3 (after the second round of purification).


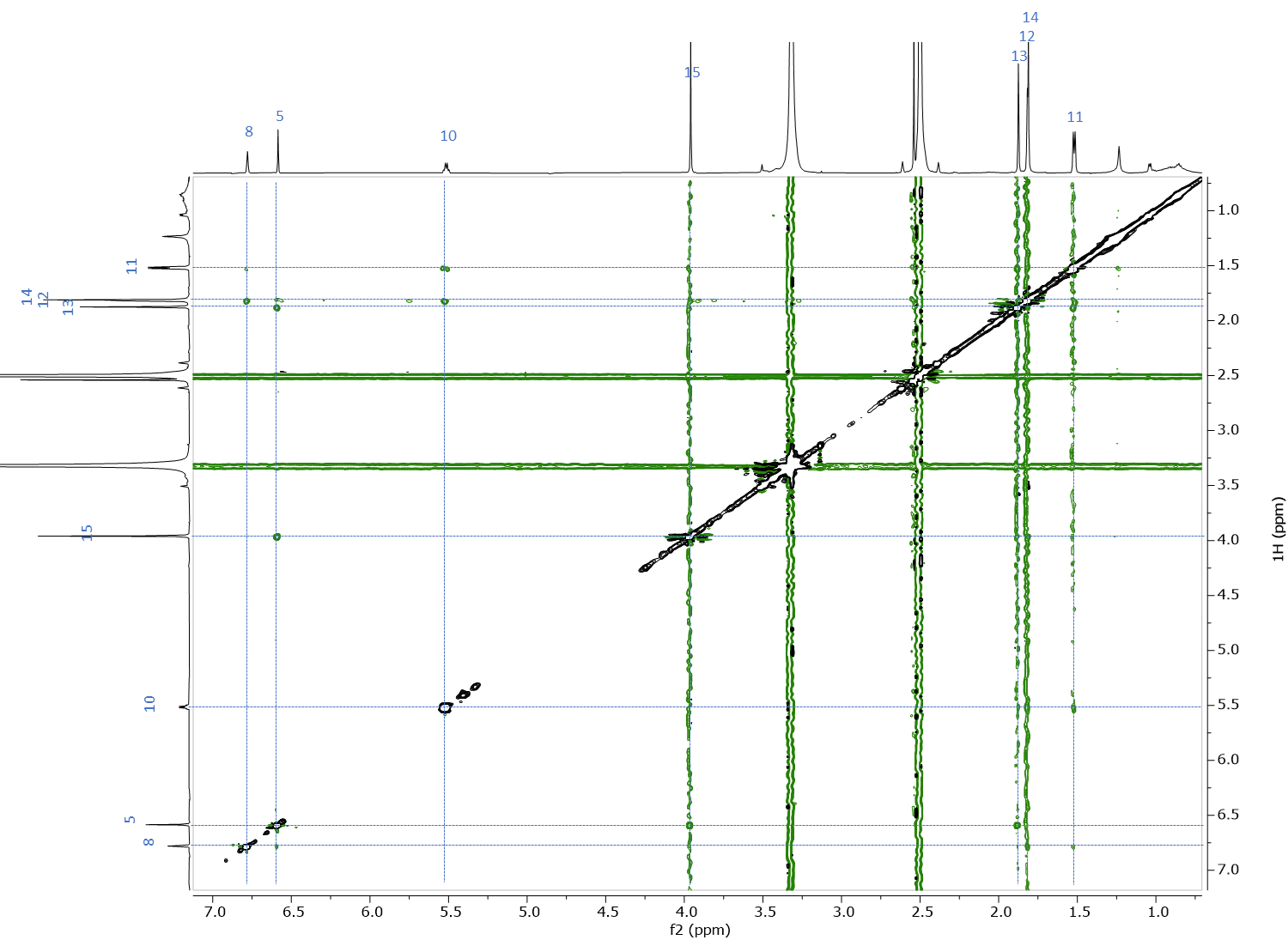


Figure S 32. ROESY of 3 (after the second round of purification).


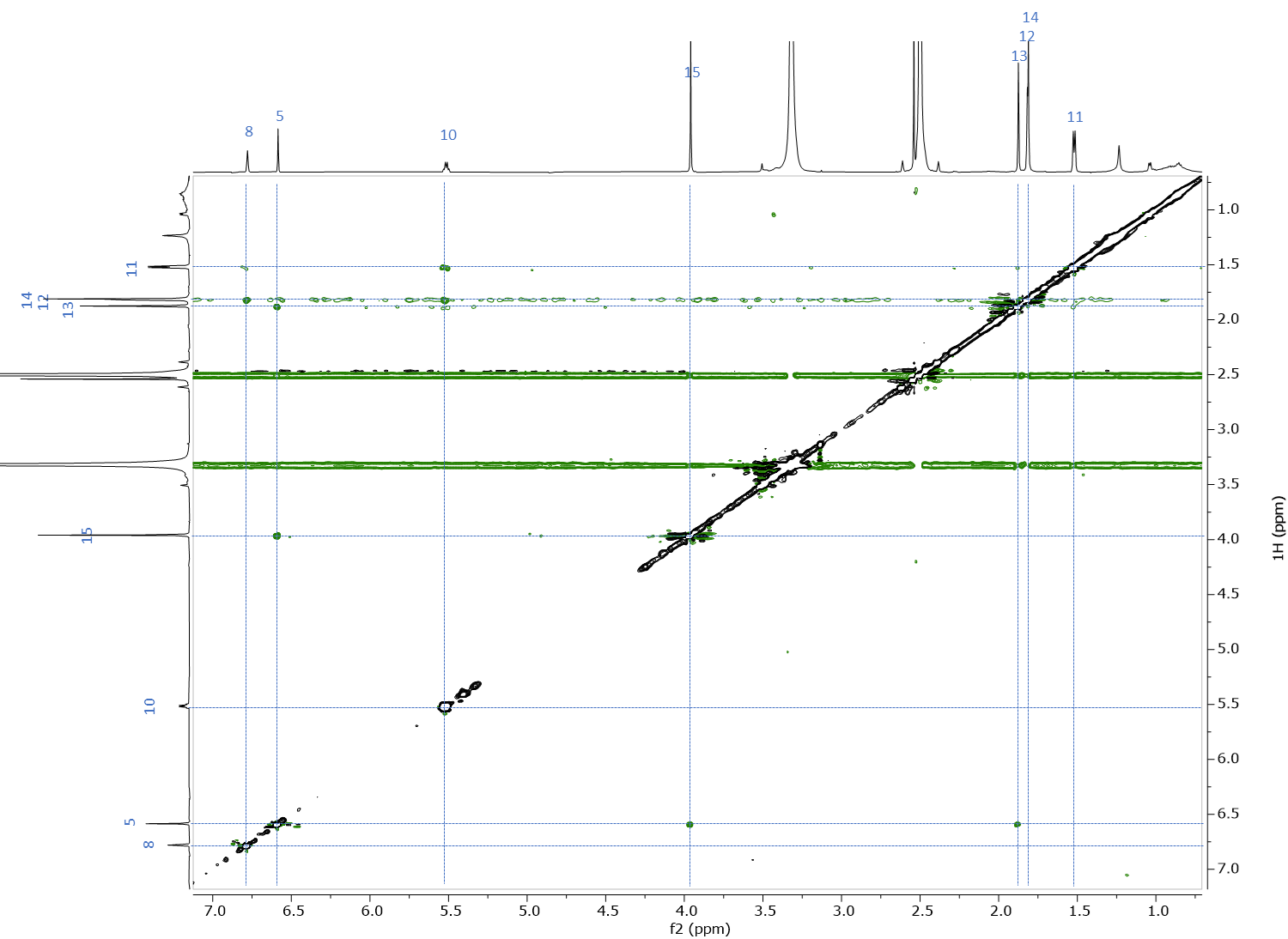


Figure S 33. ROESY (Reduced t1 noise) of 3 (after the second round of purification).

# Bioactivity screening

Figure S 34. Screening of the antibacterial activity of 2 towards five pathogenic bacteria at 125 µM.


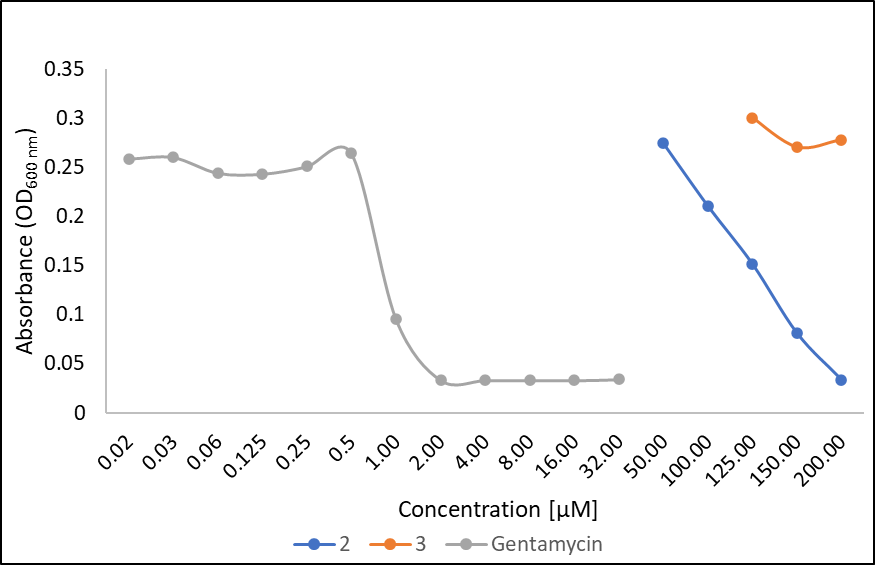


Figure S 35. Antibacterial activity of 2, 3, and gentamycin against *S. agalactiae*. Compound 2 was tested at concentrations of 50, 100, 150, and 200 μM, while 3 was tested at concentrations of 125, 150, and 200 µM. Gentamycin was used as a reference (positive control) and tested at a concentration range of 0.015–32 μM. Bacterial growth was measured at OD600 nm, and an OD value ≤ 0.05 was considered active.

## Determination of minimum inhibitory concentration (MIC) value

A MIC value of **2** against *Streptococcus agalactiae* was determined using the broth microdilution method. Compound **2** was tested at 50, 100, 150, and 200 μM concentrations. The results were plotted in a concentration/dose response curve as shown in Figure 6 of the manuscript. Additionally, a 4-parameter logistic (4PL) dose-response model was employed to analyse the relationship between the concentration of **2** and the observed response (optical density (OD) reading at 600 nm). This resulted in a sigmoidal (log-transformed) concentration-response curve. The model was fitted to the experimental data using the *drm* function from the *drc* package in R (R Core Team 2025, Ritz et al. 2015), which estimates the parameters of the 4PL function. The MIC value was calculated as the concentration (μM) corresponding to an OD of 0.05. The MIC value of **2** against *S. agalactiae* is 173 µM, as illustrated in Figure S34.


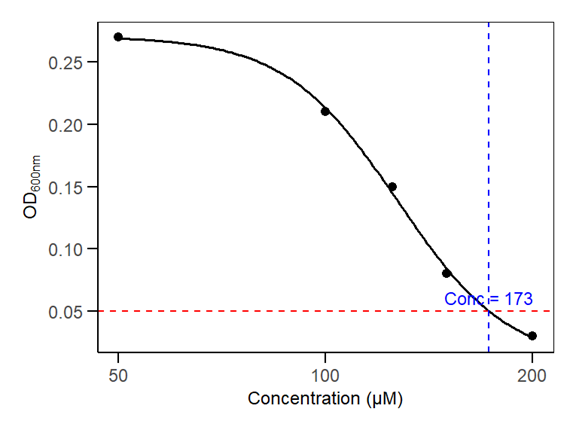


Figure S 36. Sigmoidal concentration-response curve of 2 showing the MIC value against *S. agalactiae*.

Figure S 37. Inhibition of biofilm formation of *S. epidermidis* by 2.

# References

Gargas A, & Taylor JW. Polymerase chain reaction (pcr) primers for amplifying and sequencing nuclear 18S rDNA from lichenized fungi. *Mycologia*. 1992;84(4):589-592. 10.2307/3760327

Ivanova L, Petersen D, & Uhlig S. Phomenins and fatty acids from *Alternaria infectoria*. *Toxicon*. 2010;55(6):1107-1114. 10.1016/j.toxicon.2009.12.017

Lutzoni F, Kauff F, Cox DJ et al. Assembling the fungal tree of life: Progress, classification, and evolution of subcellular traits. *Am J Bot*. 2004;91(10):1446-1480. 10.3732/ajb.91.10.1446

Pedras MSC, & Chumala PB. Phomapyrones from blackleg causing phytopathogenic fungi: Isolation, structure determination, biosyntheses and biological activity. *Phytochem*. 2005;66(1):81-87. 10.1016/j.phytochem.2004.10.011

Pedras MSC, Morales VM, & Taylor JL. Phomapyrones: Three metabolites from the blackleg fungus. *Phytochem*. 1994;36(5):1315-1318. 10.1016/S0031-9422(00)89658-2

R Core Team. (2025). R: A language and environment for statistical computing. <https://www.R-project.org/>

Rehner SA, & Buckley E. A beauveria phylogeny inferred from nuclear its and ef1-  sequences: Evidence for cryptic diversification and links to cordyceps teleomorphs. *Mycologia*. 2005;97(1):84-98. 10.3852/mycologia.97.1.84

Rehner SA, & Samuels GJ. Taxonomy and phylogeny of gliocladium analysed from nuclear large subunit ribosomal DNA sequences. *Mycol Res*. 1994;98(6):625-634. 10.1016/S0953-7562(09)80409-7

Ritz C, Baty F, Streibig JC et al. Dose-response analysis using R. *PLoS One*. 2015;10(12):e0146021. <https://doi.org/10.1371/journal.pone.0146021>

Stielow JB, Lévesque CA, Seifert KA et al. One fungus, which genes? Development and assessment of universal primers for potential secondary fungal DNA barcodes. *Persoonia*. 2015;35(1):242-263. 10.3767/003158515X689135

Tringali C, Parisi A, Piattelli M et al. Phomenins a and b, bioactive polypropionate pyrones from culture fluids of *Phoma tracheiphila*. *Nat Prod Lett*. 1993;3(2):101-106. 10.1080/10575639308043845

Vilgalys R, & Hester M. Rapid genetic identification and mapping of enzymatically amplified ribosomal DNA from several *Cryptococcus* species. *J Bacteriol*. 1990;172(8):4238-4246. 10.1128/jb.172.8.4238-4246.1990

White TJ, Bruns T, Lee S et al. Amplification and direct sequencing of fungal ribosomal RNA genes for phylogenetics. In M. A. Innis et al. (Eds.), *PCR protocols : A guide to methods and applications* (Vol. 1990;pp. 315-322). Academic Press, Inc.

Woudenberg JHC, Aveskamp MM, de Gruyter J et al. Multiple didymella teleomorphs are linked to the *Phoma clematidina* morphotype. *Persoonia*. 2009;22(1):56-62. 10.3767/003158509X427808
